# Germ Cell-Specific Gene 1-Like Protein Regulated by Splicing Factor CUGBP Elav-Like Family Member 5 and Primary Bile Acid Biosynthesis are Prognostic in Glioblastoma Multiforme

**DOI:** 10.3389/fgene.2019.01380

**Published:** 2020-02-04

**Authors:** Runzhi Huang, Zhenyu Li, Chen Li, Guanghua Wang, Penghui Yan, Li Peng, Jiaqi Wang, Xiaolong Zhu, Peng Hu, Junfang Zhang, Zhengyan Chang, Zongqiang Huang, Liming Cheng, Jie Zhang

**Affiliations:** ^1^Shanghai First Maternity and Infant Hospital, Tongji University School of Medicine, Shanghai, China; ^2^Division of Spine, Department of Orthopedics, Tongji Hospital affiliated to Tongji University School of Medicine, Shanghai, China; ^3^Tongji University School of Medicine, Shanghai, China; ^4^Department of Orthopedics, The First Affiliated Hospital of Zhengzhou University, Zhengzhou, China; ^5^Shanghai Key Laboratory of Meteorology and Health, Shanghai, China; ^6^Department of Pathology, Shanghai Tenth People’s Hospital, Tongji University School of Medicine, Shanghai, China

**Keywords:** alternative splicing, splicing factor, pathway, prognosis, glioblastoma multiforme

## Abstract

**Background:**

Alternative splicing (AS) modifies 92-94% human genes, abnormal splicing events might relate to tumor development and invasion. Glioblastoma Multiforme (GBM) is a fatal, invasive, and malignant tumor in nervous system. The recurrence and development leads to poor prognosis. However, few studies have focused on AS in GBM.

**Methods:**

RNA-seq and Alternative splicing events (ASEs) data of GBM samples were downloaded from The Cancer Genome Atlas (TCGA) and TCGASpliceSeq databases, respectively. Firstly, the Cox regression analysis was utilized to identify the overall survival splicing events (OS-SEs). Secondly, a multivariable model was applied to access the prognostic value of risk score. Then, we constructed a co-expressed network between splicing factors (SFs) and overall survival alternative splicing events (OS-SEs). Additionally, to explore the relationship between the potential prognostic signaling pathways and OS-SEs, we constructed a network between these pathways and OS-SEs. Ultimately, to better explain the results, validations from multi-dimension platforms were applied.

**Results:**

In the first step, 1,062 OS-SEs were selected by Cox regression. Then, 11 OS-SEs were integrated in a multivariate model by Lasso regression. The area under the curve (AUC) of receiver operator characteristic (ROC) curve was 0.861. In addition, the risk score generated from the multivariate model was confirmed to be an independent prognostic factor (P < 0.001). What's more, in the network of SFs and ASEs, CELF5 significantly regulated GSG1L|35696|AP and GSG1L|35698|AP (P < 0.001, R = 0.511 and = -0.492). Additionally, GSG1L|35696|AP (P = 0.006) and GSG1L|35698|AP (P = 0.007) showed a significant relationship with cancer status. Eventually, KEGG pathways related to prognosis of GBM were selected by GSVA. The primary bile acid synthesis (P < 0.001, R = 0.420) was the significant pathway co-expressed with Germ Cell-Specific Gene 1-Like Protein (GSG1L).

**Conclusions:**

Based on the comprehensive bioinformatics analysis, we proposed that aberrant splicing factor CUGBP Elav-like family member 5 (CELF5) significantly, positively and negatively, regulated ASE of GSG1L, and the primary bile acid synthesis pathway might play an important role in tumorigenesis and prognosis of GBM.

## Introduction

Alternative splicing (AS) is a common process for producing multiply mature RNA from pre-RNA, and it can contribute to the modification of 92-94% human genes (Wang et al., 2008). Alternative splicing events (ASEs) are regulated by splicing factors (SFs) (Lee and Abdel-Wahab, 2016). Abnormal splicing can improve tumor development by changing metabolism (Kozlovski et al., 2017). In addition, the regulatory relationship between OS-SE and SF can be involved in the tumor biology process and be regarded as the predictor for prognosis in certain cancers (Yang et al., 2019).

Glioblastoma multiforme (GBM) is the most fatal and invasive malignant tumor in the nervous system. The World Health Organization (WHO) defines GBM as the IV grade glioma. It is reported that there are three new cases of GBM per 100,000 people every year, and the two-year and five-year survival rate are 26-33% and 4-5%, respectively (Batash et al., 2017). Generally, the common effective treatments for GBM are surgery, chemotherapy, and radiation. However, due to high incidences of recurrence and progression, GBM patients often have poor prognosis (Alifieris and Trafalis, 2015; Zhou et al., 2018b). Among the reasons for poor prognosis, transcriptome alteration has been proved to be of great importance in the process of tumorigenesis and progression in rat models. However, to date, most previous studies of GBM have focused on genomic and transcriptome levels, and post-transcriptional regulatory mechanisms have been largely neglected. There are few remarkable biomarkers for prognosis, and no studies have been devoted to exploring the relationship between SFs and ASEs in GBM, which might also play a role in the tumorigenesis and prognosis in GBM.

In the current study, we utilized a Cox regression analysis to identify the overall survival of splicing events (OS-SEs) and constructed a multivariate prognosis model. In addition, the risk score generated from the multivariate model was confirmed to be an independent prognostic factor. We constructed a co-expressed network between SFs and OS-SEs. Meanwhile, to explore the relationship between the potential prognostic signaling pathways and OS-SEs, we constructed a network between these pathways and OS-SEs. Ultimately, we found a regulatory network related to the cancer status and prognosis in GBM, in which the interaction SFs and ASEs were included.

## Method

### Data Extraction

This study was approved by the Ethics Committee of Tongji University School of Medicine. Clinical information and gene expression profiling in 599 primary GBM samples were available from The Cancer Genome Atlas (TCGA) database (https://tcga-data.nci.nih.gov/tcga/). In addition, Percent Spliced In (PSI) values of ASEs more than 75% in primary GBM were downloaded from TCGASpliceSeq database (https://bioinformatics.mdanderson.org/TCGASpliceSeq/) (Ryan et al., 2016). Then, an UpSet plot was developed to demonstrate genes processed by splicing events and patterns of splicing events in GBM.

### OS-SEs Identification

In data preprocessing, the missing data in PSI values was supplemented by K-Nearest Neighbor algorithm (k = 10, rowmax = 0.5, colmax = 0.8, maxp = 1500, rng.seed=362436069). Next, the ASEs with percent spliced in (PSI) values were filtered. ASEs with mean PSI values of less than 0.05 and/or standard derivations of PSI values less than 0.01 were excluded. Samples from patients without demographic information and follow-up records were also excluded. Next, the rest data were analyzed by univariate Cox regression to identify the OS-SEs. The UpSet plot was applied to demonstrate the result. In the third step, a volcano plot was used to illustrate the prognostic of OS-SEs, and the x-axis and y-axis represented z-score and -log10 (P value) in the volcano plot, respectively. Moreover, the top 20 OS-SEs in each splicing pattern: Alternate acceptor site (AA), Alternate donor site (AD), Alternate promoter (AP), Alternate terminator (AT), Exon skip (ES), Mutually exons (ME), and Retained intron (RI) was selected and demonstrated in bubble plots.

### Multivariate Model Construction and Independent Prognostic Factor Identification

To avoid over fitness in the multivariate model, Lasso regression was utilized to screen the most significant OS-SEs in each splicing pattern. Then, the selected OS-SEs were integrated into the multivariate model. Receiver operator characteristic (ROC) curve was applied to access the efficiency of the prognosis model. The risk score of each sample for predicting the prognosis was calculated by the following formula:

$$Risk\;score_{i}=\beta_{\mathrm{OS}-\text{SE}1}\times {\mathrm{PSI}}_{\mathrm{OS}-\text{SE}1}+\beta_{\mathrm{OS}-\text{SE}2}\times {\mathrm{PSI}}_{\mathrm{OS}-\text{SE}2}+\cdots \cdots +\beta_{\mathrm{OS}-\mathrm{SEn}}\times PSI_{\mathrm{OS}-\mathrm{SEn}}$$

In the formula, “n” represented the number of OS-SEs in the multivariate model, “β” represented the regression coefficient of each OS-SE from multivariate model, “*Risk score_i_*” represented the risk score of No. “i” patient. Further, the median was selected to divide the risk score into high and low. Moreover, the Kaplan-Meier curve was utilized to evaluate the relationship between risk score and survival probability.

### Independent Prognostic Analysis

The univariate and multivariable Cox regression analyses were utilized to access the prognostic value of risk score generated from the multivariate model. The demographics and clinical information including age, gender, and cancer status were used for model correction.

### SF and OS-SEs Network Construction

Based on SpliceAid2 database (Piva et al., 2009), a dataset with 390 SFs was downloaded. Next, the relationships between 390 SFs and OS-SEs were analyzed by Pearson correlation. Relationships with a correlation coefficient > 0.400 and P < 0.001 (Pearson correlation analysis) were selected as significant SFs-OS-SEs for next step. Finally, the network including the significantly co-expressed SFs and OS-SEs was developed by Cytoscape (3.7.1) (Shannon et al., 2003).

### OS-SEs Related to Cancer Status and Co-Expressed With Pathways Identification

The Mann-Whitney-Wilcoxon test and Kruskal-Wallis test were applied to identify the relationship between OS-SEs and GBM status, and the Beeswarm plots were utilized to illustrate the significance of the relationship.

Gene Set Variation Analysis (GSVA) was utilized to select the differential expression Kyoto Encyclopedia of Genes and Genomes (KEGG) pathways in GBM. Then, prognostic KEGG pathways were identified by univariate Cox analysis based on GSVA. The Pearson analysis was applied to identify the correlation between cancer status-related OS-SEs and prognostic KEGG pathways. Based on the result, significantly co-expressing KEGG pathways and OS-SEs were selected.

### Multidimensional Online Verification

In order to increase the reliability of the results produced by *in silico* analysis, multidimensional validation from other platforms was performed. Pathway Card (https://pathcards.genecards.org/) was utilized to find the top 5 genes related to the selected KEGG pathway. Then, The Human Protein Altas (Uhlen et al., 2015) and Genotype-Tissue Expression (GTEx) (Consortium, 2015) showed the key genes and proteins expression levels in normal tissue. PROGgeneV2 (Goswami and Nakshatri, 2014), Gene Expression Profiling Interactive Analysis (GEPIA) (Tang et al., 2017), UCSC xena (Goldman et al., 2015), SurvExpress (Aguirre-Gamboa et al., 2013), UALCAN (Chandrashekar et al., 2017), Linkedomics (Vasaikar et al., 2018), cBioportal (Cerami et al., 2012) and Oncomine (Rhodes et al., 2004) showed the genes expression level at the tissue level in GBM. Further, Cancer Cell Line Encyclopedia (CCLE) (Ghandi et al., 2019) was applied to show the gene expression at the cellular level in GBM. Eventually, String (Snel et al., 2000) illustrated the interaction network of SF, OS-SE, and the potential pathway. Additionally, an independent dataset named Chinese Glioma Genome Atlas (CGGA) (Hu et al., 2019) was used for external validation of key genes.

### Statistical Analysis

A two-tailed P-value < 0.05 was considered as statistically significant. In order to control the size of the SF regulation network, correlation coefficient > 0.400 and P < 0.001 in Pearson correlation analysis were selected as a more stringent screening criteria to screen for co-expression patterns between SFs and OS-SEs. All statistical analysis was performed by R version 3.6.1 (Institute for Statistics and Mathematics, Vienna, Austria; www.r-project.org) (Package: impute, UpSetR, ggplot2, rms, glmnet, preprocessCore, forestplot, survminer, survivalROC, beeswarm).

## Result

### OS-SEs Identification

**Figure 1:**
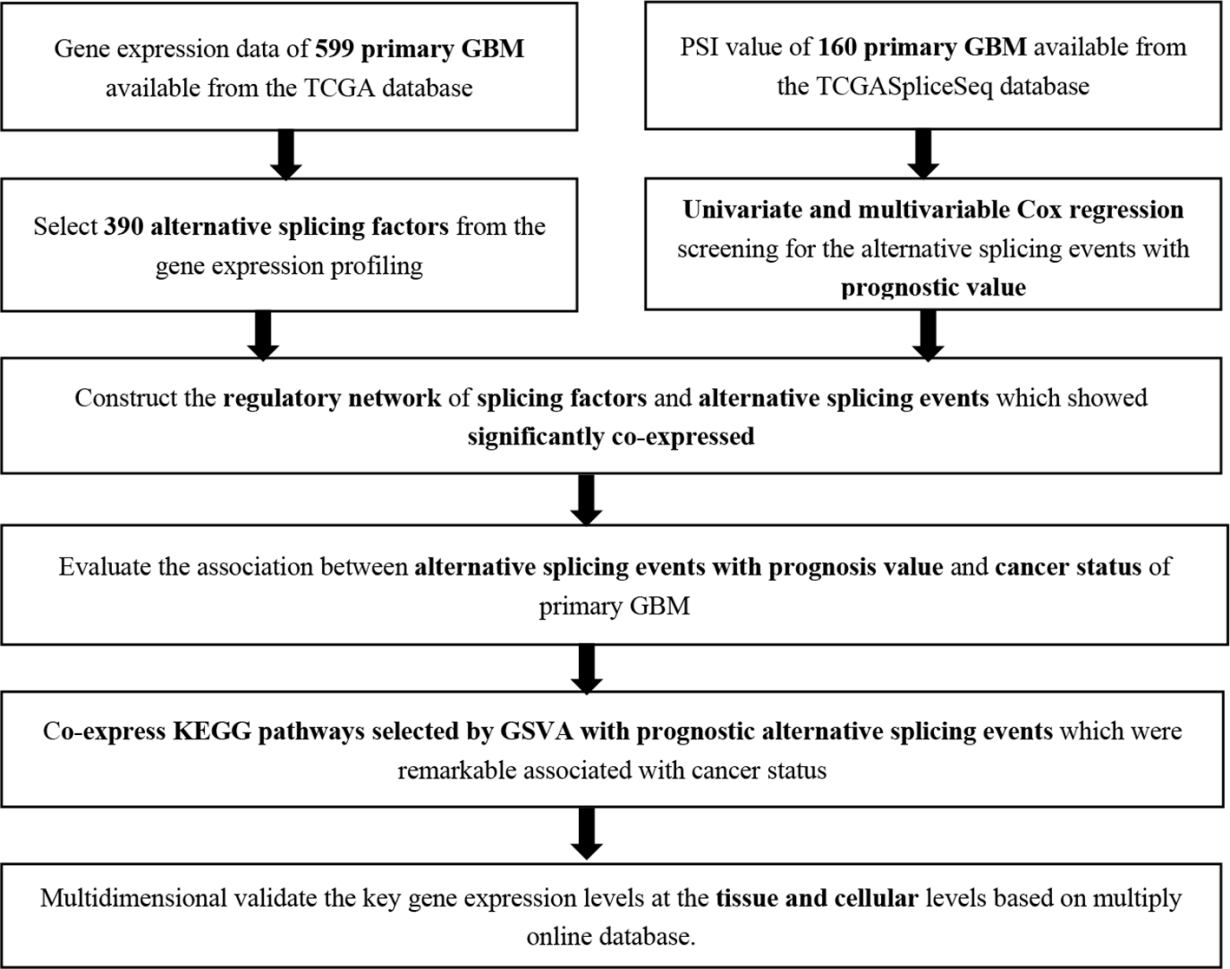
The flow chart of analysis method.

**Figure 2:**
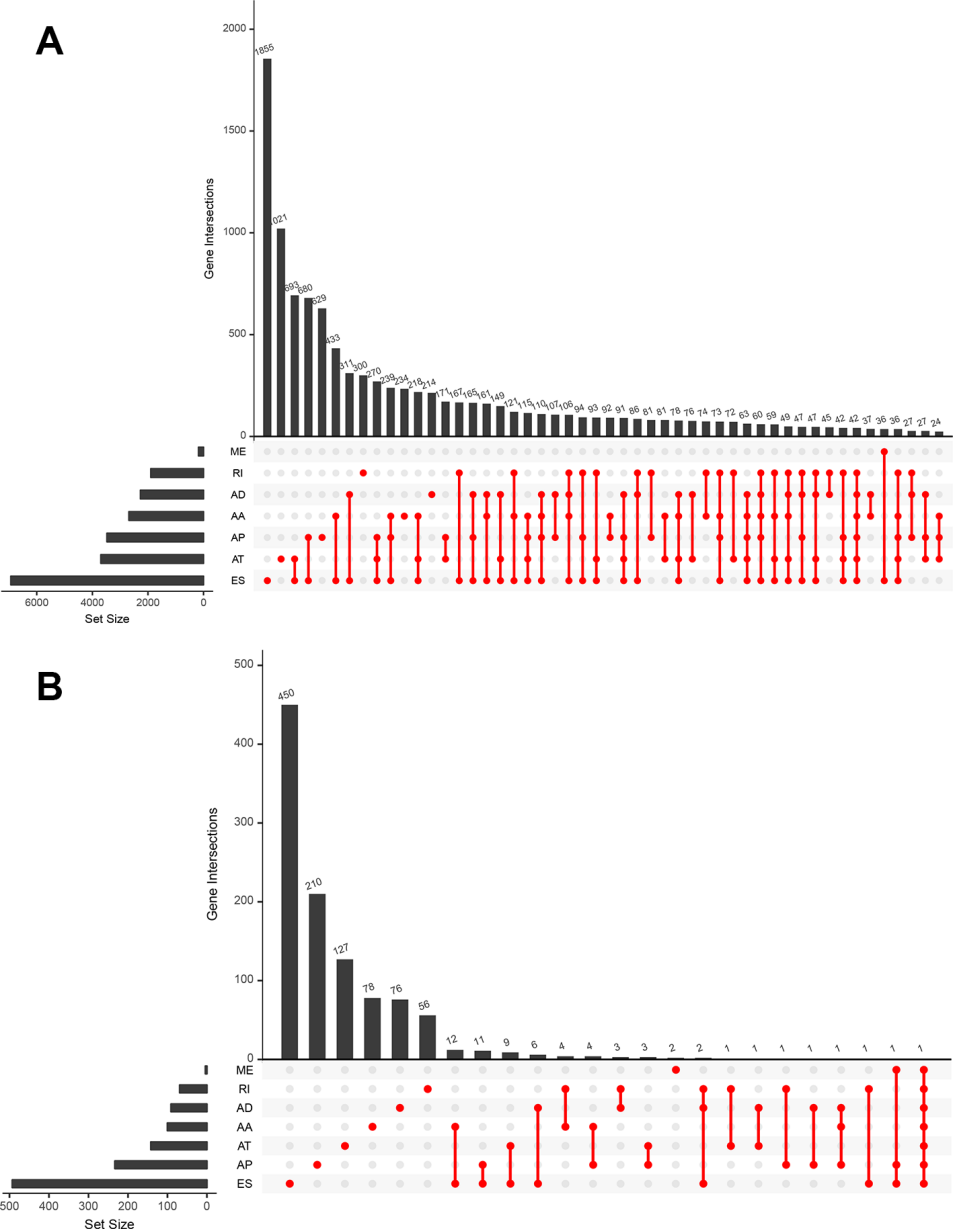
The UpSet plots of alternative splicing event patterns and genes **(A)** and alternative splicing event patterns and genes which related to survival **(B)**. The horizontal bar graph represented splicing patterns, vertical bar graph represented genes processed alternative splicing events and the red dots represent the intersection point of splicing patterns and genes processed alternative splicing events. ME, Mutually exons; AD, Alternate donor site; RI, Retained intron; AA, Alternate acceptor site; AP, Alternate promoter; AT, Alternate terminator; ES, Exon skip.

**Figure 3:**
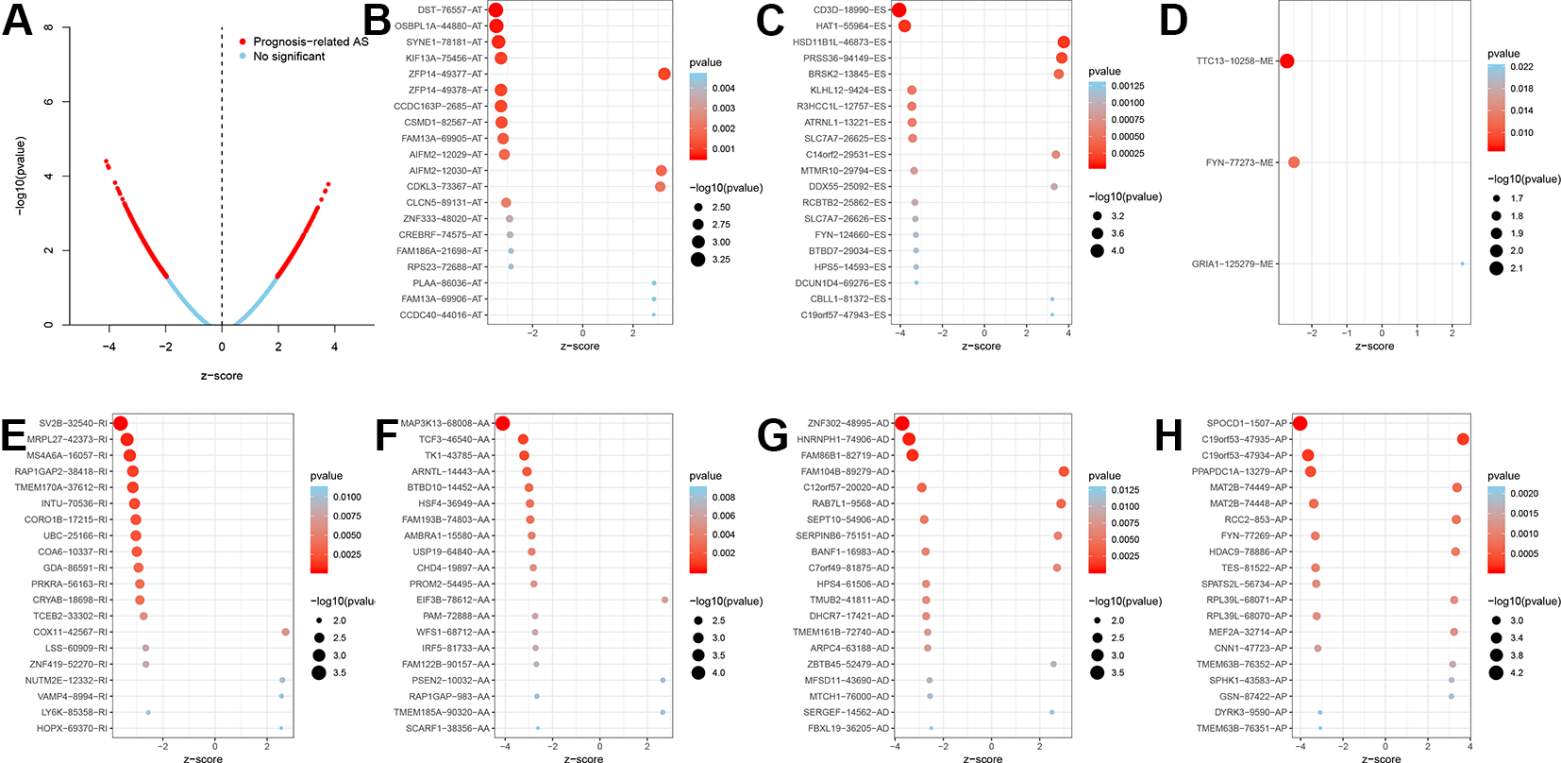
The Volcano plot of alternative splicing events to divide significant and non-significant overall-survival-associated splicing events **(A)**, red dots represented significant prognosis-related while blue dots represent non-significant. The bubble plots of the top 20 overall-survival-associated splicing events **(B**–**H)**. AS, Alternative splicing; AT, Alternate terminator; ES, Exon skip; ME, Mutually exons; RI, Retained intron; AA, Alternate acceptor site; AD, Alternate donor site; AP, Alternate promoter.

The analysis process was illustrated in **Figure 1**. Gene expression profiling of 599 primary GBM samples available from TCGA database was analyzed. **Table 1** summarizes the baseline information of the patients. We defined a pattern to show each ASE: the gene name, the TCGASliceSeq database AS ID of each ASE, and the splicing pattern were integrated like GSG1L|35696|AP, so the GSG1L was the gene-symbol, 35696 was the AS ID, and AP was the splicing pattern. A total of 21,407 ASEs in 10,101 genes were identified in patients with GBM, including 2,738 AAs in 2,505 genes, 2,301 ADs in 2,098 genes, 3,524APs in 3,285 genes, 3,728 ATs in 3,517 genes, 6,961 ESs in 6,789 genes, 233 MEs in 36 genes, and 1,922 RIs in 1,727 genes. Besides ASE genes and splicing patterns in all primary GBM shown in the UpSet plot, several genes correlated to multiply the splicing pattern (**Figure 2A**). OS-SEs identified by Cox regression are shown in the UpSet plot (**Figure 2B**). Generally, ES was the most significant splicing pattern in GBM. Additionally, the volcano plot of ASEs demonstrated the significant and non-significant OS-SEs (**Figure 3A**). Moreover, DST|76557|AT, CD3D|18990|ES, TTC13|10258|ME, SV2B|32540|RI, MAP3K13|68008|AA, ZNF302|48996|AD, and SPOCD1|1507|AP were the most significant of the top 20 OS-SEs in each splicing pattern in each bubble plot (**Figures 3B–H**).

**Table 1:** Baseline information of 599 patients with Glioblastoma Multiforme available from the TCGA database.

Variables	Total Patients (N = 599)
**Age, years**	
Mean ± SD	57.83 ± 14.41
Median (Range)	59.00 (10 - 89)
**Gender**	
Female	230 (38.40%)
Male	366 (61.10%)
Unknown	3 (0.50%)
**Cancer status**	
With tumor	507 (84.64%)
Tumor free	28 (4.67%)
unknow	64 (10.68%)

SD, Standard deviation.

### Multivariate Model Construction

**Figure 4:**
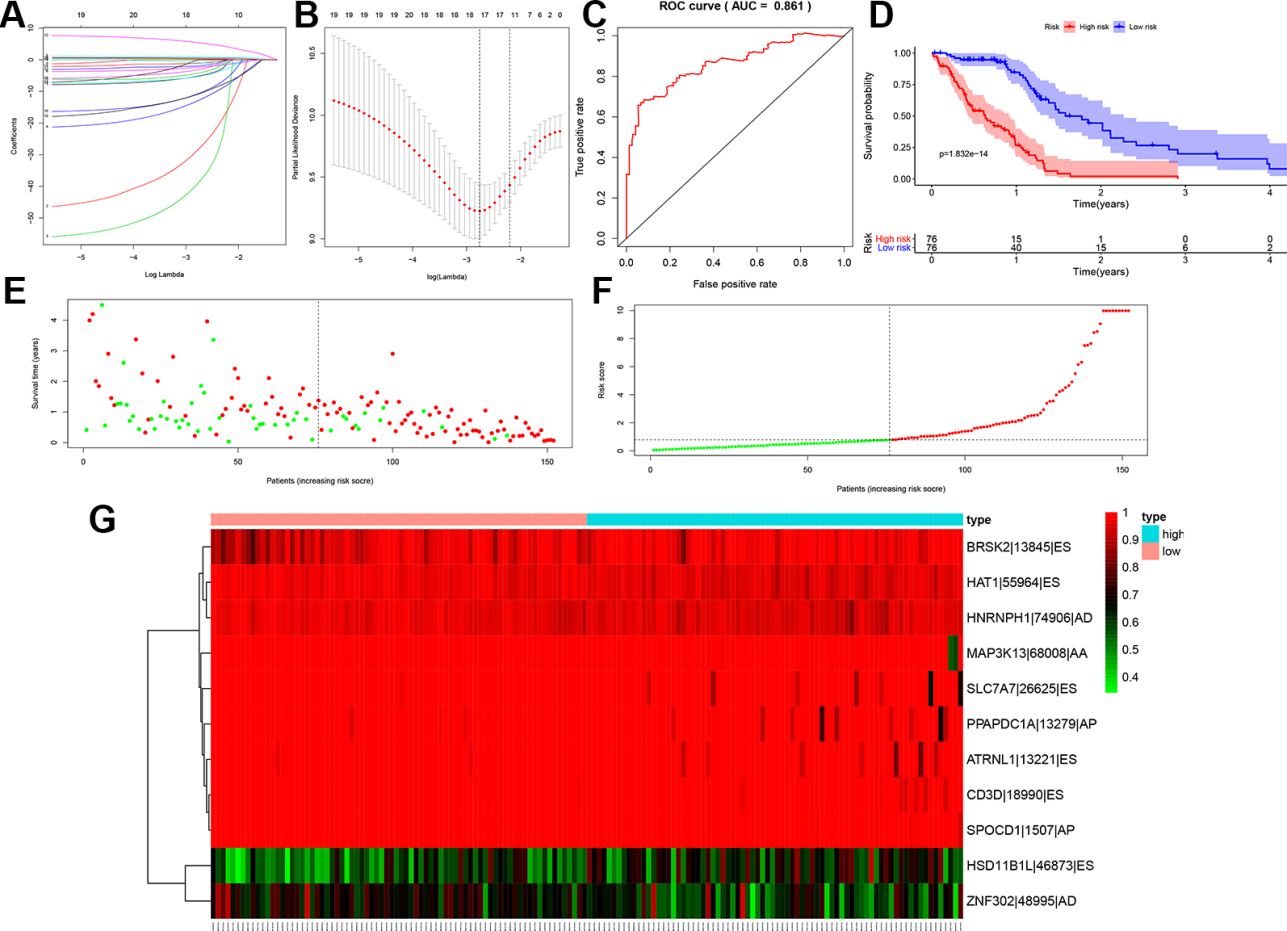
Lasso regression for the top 20 overall-survival-associated splicing events with the smallest P values **(A**, **B)**, the receiver operator characteristic curve to access the prognosis model **(C)**, the Kaplan-Meier curve to identify the efficacy of risk score's in overall survival **(D)**, the high and low risk score group in scatterplot **(E)** and risk plot **(F)** for each sample of GBM based on the profiling from TCGA database, the heatmap to illustrated each overall-survival-associated splicing event's expression level screened by Lasso regression screened **(G)**. (AUC = 0.861). ES, Exon skip; AT, Alternate terminator; RI, Retained intron; AP, Alternate promoter.

Before building the prognosis model, the OS-SEs screened by Lasso regression were utilized to avoid over-fitness. Then, BRSk2|13845|ES, HAT1|55964|ES, HNRNPH1|74906|AD, MAP3K13|68008|AA, SLC7A7|26625|ES, PPAPDC1A|13279|AP, ATRNL1|13221|ES, CD3D|18990|ES, SPOCD1|1507|AP, HSD11B1L|46873|ES, and ZNF302|48995|AD were integrated into the multivariate model (**Figures 4A, B**). The area under the curve (AUC) of the ROC curve was 0.861 (**Figure 4C**). In addition, to divided the high and low group, the risk score median was set as 0.780. Accessed by the Kaplan-Meier curve, the prognostic efficiency of the risk score was significant (P < 0.001) (**Figure 4D**). The relationship of risk score and clinical information was shown in the scatter plot and risk curve (**Figures 4E, F**). In addition, the red and green dots represented high and low risk in the scatterplot and risk curve. Expression level of OS-SEs was illustrated in the heatmap. BRSk2|13845|ES, HAT1|55964|ES, HNRNPH1|74906|AD, MAP3K13|68008|AA, SLC7A7|26625|ES, PPAPDC1A|13279|AP, ATRNL1|13221|ES, CD3D|18990|ES, and SPOCD1|1507|AP expressed higher than the high risk group, while HSD11B1L|46873|ES and ZNF302|48995|AD expressed lower than the low risk group (**Figure 4G**). Red and blue bars represent high and low risk groups in the heatmap.

### Independently Prognostic Analysis

**Figure 5:**
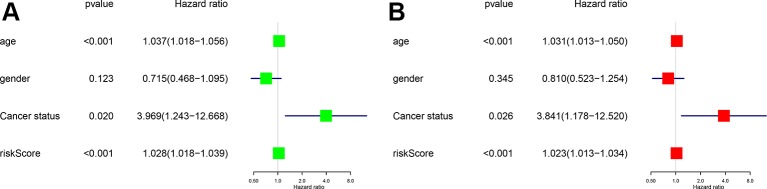
Univariate **(A)** and multivariate cox regression analysis **(B)** Forest plots. Green for univariate and red for multivariate.

To access the independent prognostic value of the risk score generated from the multivariable model, the univariate (HR = 1.028, 95%CI (1.018-1.039), P < 0.001) (**Figure 5A**) and multivariate (HR = 1.023, 95%CI (1.013-1.034), P < 0.001) Cox regression analyses (**Figure 5B**) were applied. Some other clinical variables, such as age and cancer status, were also significant. Based on the univariate and multivariate Cox regression analyses results, the risk score generated from the multivariate model was confirmed to be an independent prognostic factor in GBM.

### OS-SEs and SFs Network Construction and Cancer Status-Related Analysis

**Figure 6:**
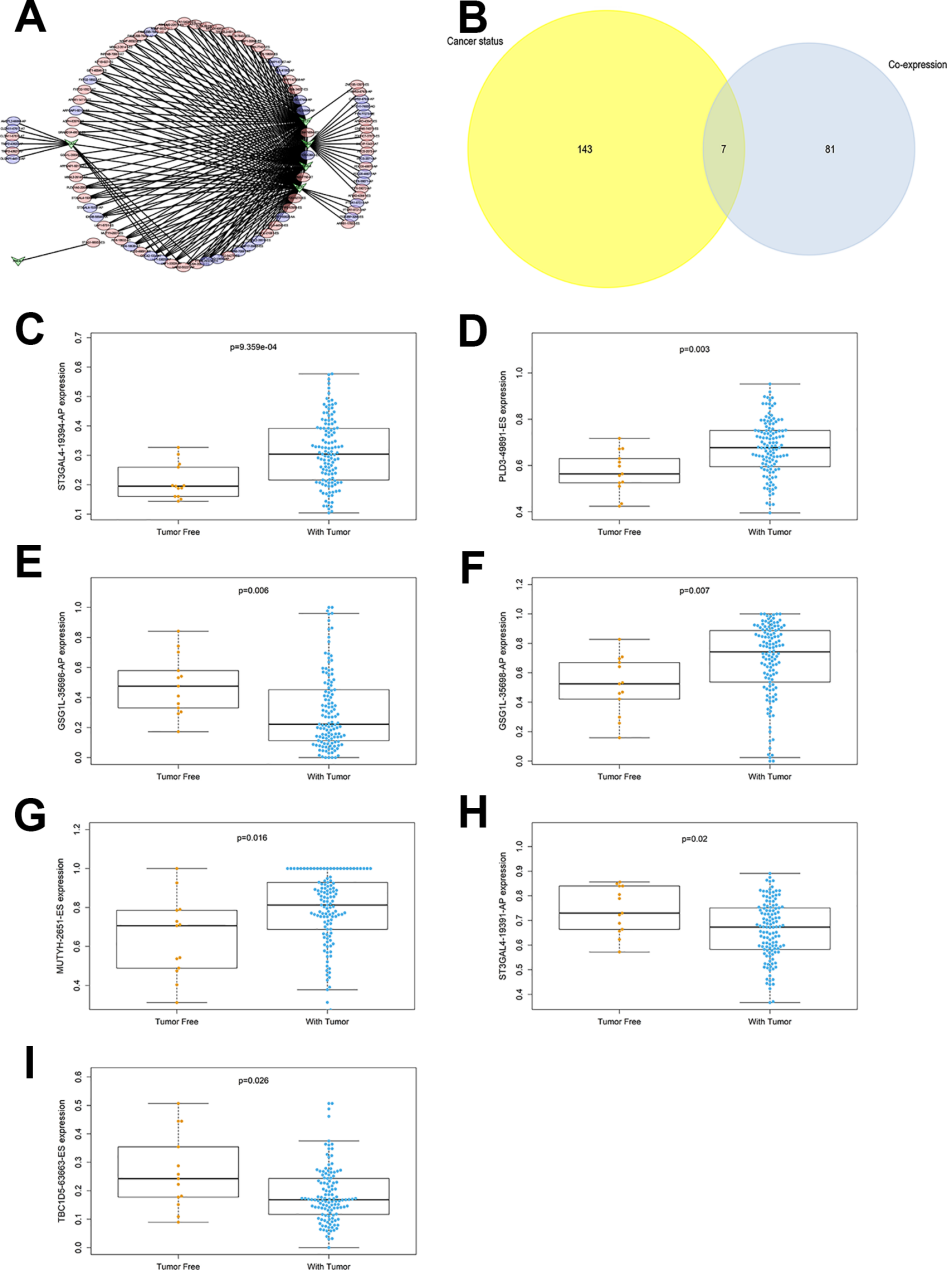
The network constructed for co-expressed splicing factors and overall-survival-associated splicing events **(A)**, arrows represented SFs, the red and blue ellipses represented high and low risk of OS-SEs. The venn plot to show the relationship between overall-survival-associated splicing events and cancer status **(B)**, the bar plot to show the relationship between ST3GAL4|19394|AP and cancer status **(C)**, the bar plot to show the relationship between PLD3|49891|ES and cancer status **(D)**, the bar plot to show the relationship between GSG1L|35696|AP and cancer status **(E)**, the bar plot to show the relationship between GSG1L|35698|AP and cancer status **(F)**, the bar plot to show the relationship between MUTYH|2651|ES and cancer status **(G)**, the bar plot to show the relationship between ST3GAL4|19391|AP and cancer status **(H)**, the bar plot to show the relationship between TBC1D5|63663|ES and cancer status **(I)**. AT, Alternate terminator; AD, Alternate donor site; ES, Exon skip; AP, Alternate promoter.

In the network of SFs and OS-SEs, arrows represented SFs, and the red and blue ellipses represented high and low risk of OS-SEs, respectively. Further, CELF3 significantly regulated GSG1L|35696|AP and GSG1L|35698|AP (P < 0.001, R = 0.447 and R = -0.426), CELF5 significantly regulated GSG1L|35696|AP and GSG1L|35698|AP (P < 0.001, R = 0.511 and R = -0.492), and ELAVL3 significantly regulated ST3GAL4|19391|AP and ST3GAL4|19394|AP (P < 0.001, R = 0.521 and R = -0.548) (**Figure 6A**). Seven OS-SEs were shown to be cancer status-related in a Venn plot (**Figure 6B**). Besides, ST3GAL4|19394|AP was related to cancer status (P < 0.001), PLD3|49891|ES was related to cancer status (P = 0.003), GSG1L|35696|AP was related to cancer status (P = 0.006), GSG1L|35698|AP was related to cancer status (P = 0.007), MUTYH|2651|ES was related to cancer status (P = 0.016), ST3GAL4|19391|AP was related to cancer status (P = 0.020), and TBC1D5|63663|ES was related to cancer status (P = 0.026) (**Figures 6C–I**).

### OS-SEs Related to Status and Survival-Related Pathways Co-Analysis

**Figure 7:**
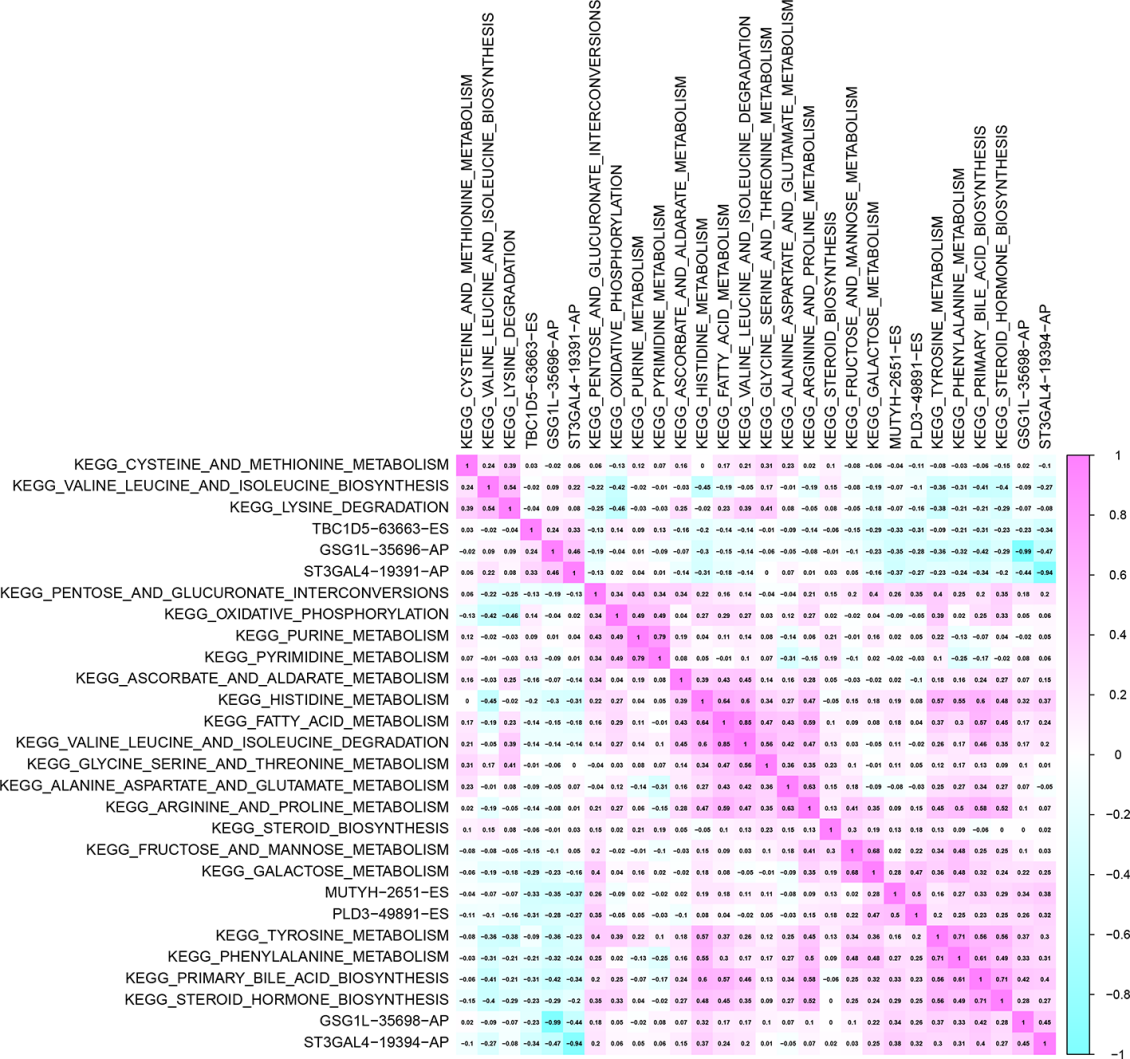
The heatmap of co-expression overall-survival-associated splicing events related to cancer status and Kyoto Encyclopedia of Genes and Genomes (KEGG) pathways selected by Gene Set Variation Analysis (GSVA).

185 prognostic KEGG pathways were identified based on GSVA and univariate Cox analysis. The correlation of OS-SEs and KEGG pathways were analyzed by Pearson analysis (**Figure 7**). Combined with the results of the significant relationship in the SFs and OS-SEs network, CELF5 (SF) was considered as a remarkable marker related to GSG1L (OS-SEs) (P < 0.001, R = 0.511 and -0.492), and the most five significant pathways of GSG1L were primary bile acid synthesis (P < 0.001, R = 0.420) , tyrosine metabolism (P < 0.001, R = 0.360), phenylalanine metabolism (P < 0.001, R = 0.320), histidine metabolism (P < 0.001, R = 0.300), and steroid hormone biosynthesis (P < 0.001, R = 0.290), respectively.

**Figure 8:**
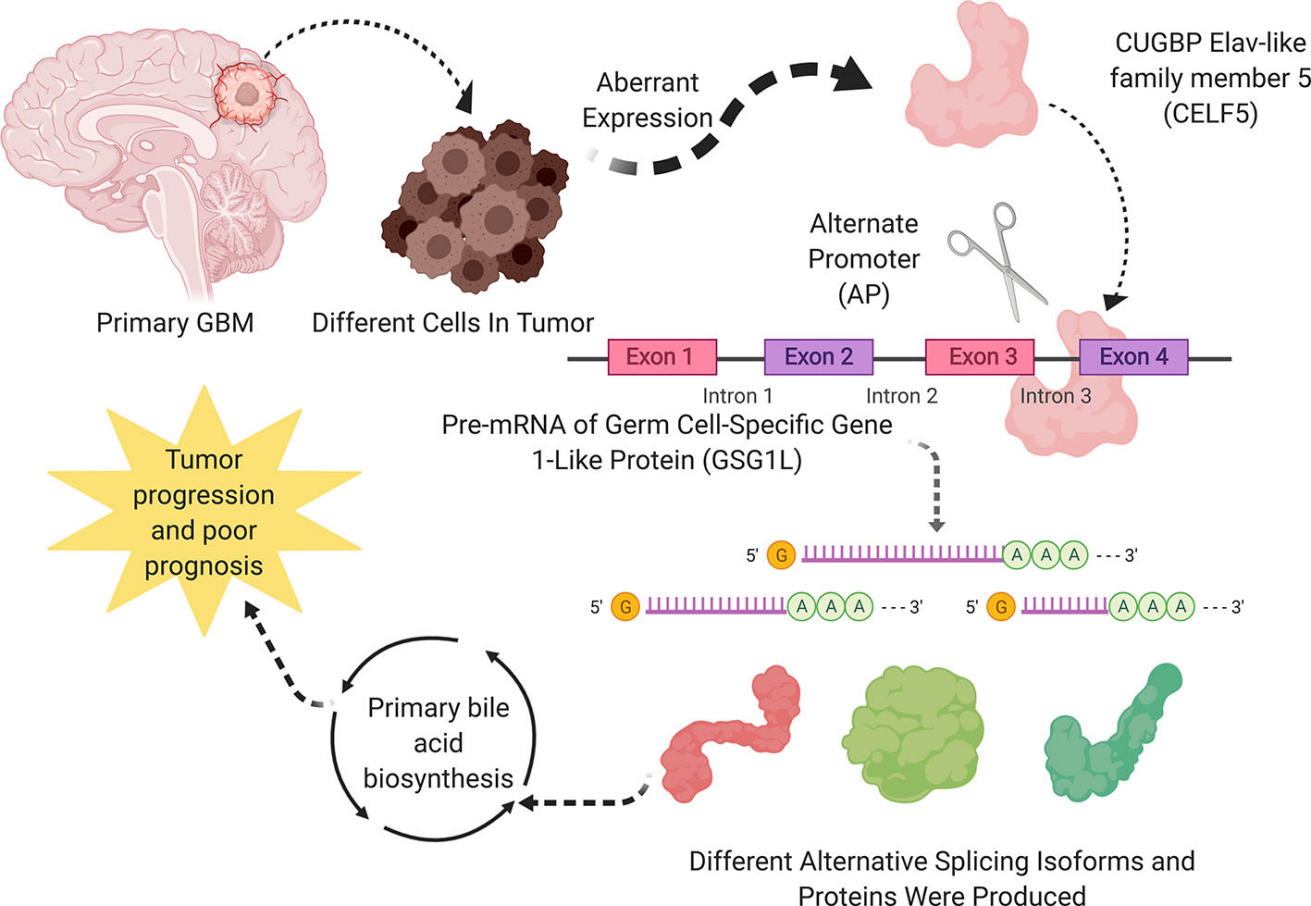
A speculatively schematic diagram of this scientific hypothesis including the most significant SF (CLEF5), OS-SEs (GSG1L|35698|AP, GSG1L|35696|AP) and downstream pathway (Primary bile acid synthesis).

All in all, the most significant SF, OS-SEs, and downstream pathway were CLEF5, GSG1L|35698|AP, GSG1L|35696|AP and primary bile acid synthesis, respectively. A schematic diagram of this scientific hypothesis is shown in **Figure 8**.

### Multidimensional Validation

With the aim of minimizing the bias of results above, multi-platforms validations were performed. Pathway Card showed that AMACR, AKR1D1, CYP27A1, CYP46A1, and CH25H were the top five genes in primary bile acid synthesis. The detail results of the human protein atlas (**Figure S1**), PROGgeneV2 (**Figure S2**), GEPIA (**Figure S3**), UCSC xena (**Figure S4**), SurvExpress (**Figure S5**), UALCAN (**Figure S6**), Linkedomics (**Figure S7**), cBioportal (**Figure S8**, **Table S1**). Oncomine (**Figure S9**), CCLE (**Figure S10**), and String (**Figure S11**) were shown in **Supplementary Material**.

First of all, the expression levels of CELF5, GSG1L, AMACR, AKR1D1, CYP27A1, CYP46A1, and CH25H in multiple databases were summarized in **Table S2**. CELF5, CYP27A1, and CYP46A1 were high-expressed, while AMACR and CH25H were low-expressed in normal cerebral cortex in the brain (**Figure S1**). GSG1L, AMACR, and CYP27A1 were high-expressed, while CELF5 was low-expressed in tumors at tissue level (**Figures S2**-**S9**). CELF5 was low-expressed in tumor cell line; AMACR, CYP27A1, CYP46A1, and CH25H were high-expressed in tumor cell line in CCLE (**Figure S10**). The interaction PPI network of CELF5, GSGS1L, AMACR, AKR1D1, CYP27A1, CYP46A1, and CH25H in the String was shown in **Figure S11**.

**Figure 9:**
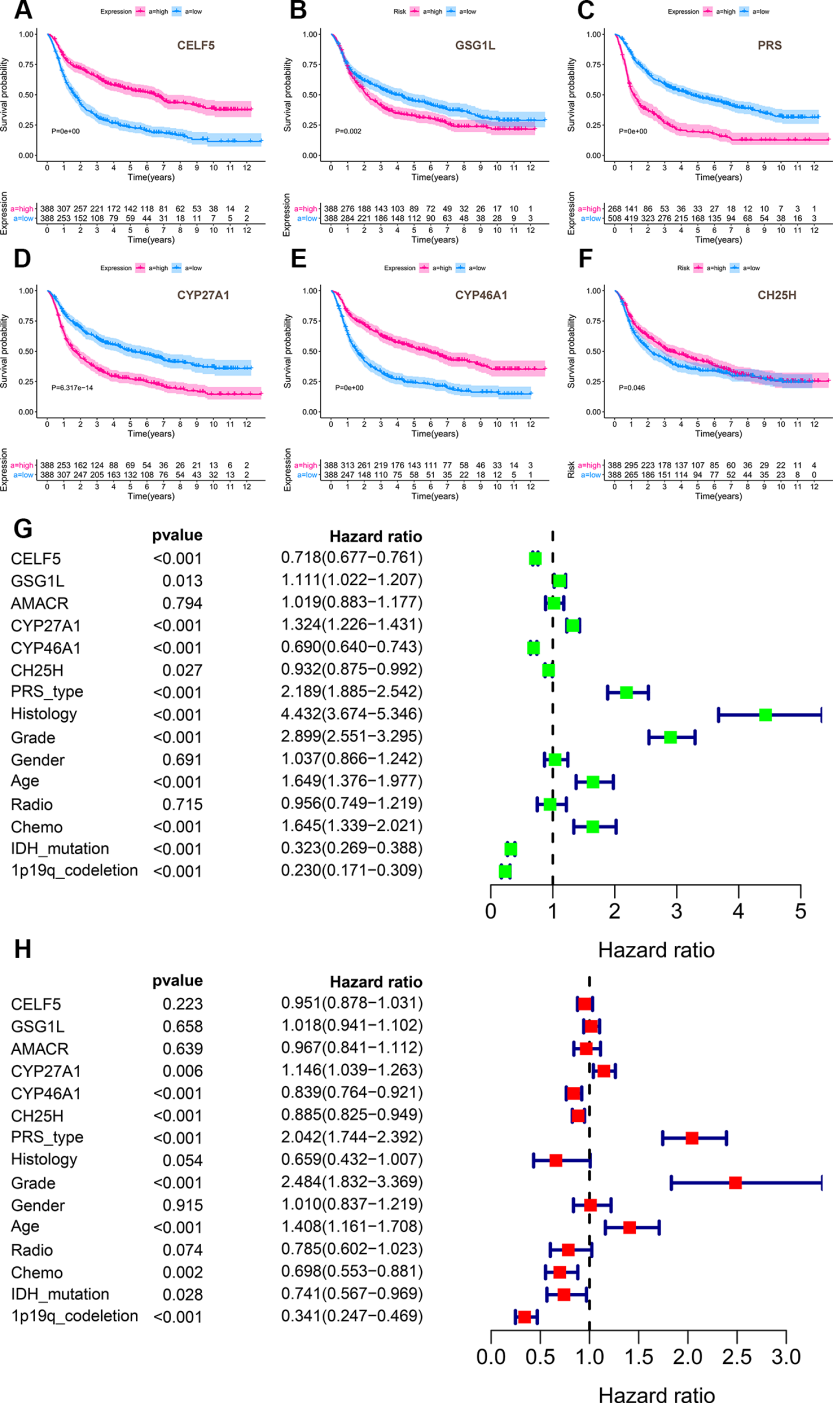
The results of external validation of CELF5, GSG1L, AMACR, CYP27A1, CYP46A1 and CH25H using an independent dataset named Chinese Glioma Genome Atlas (CGGA). Analysis based on 1,018 Chinese glioma patients suggested that CELF5 (P < 0.001), GSG1L (P = 0.002), AMACR (P < 0.001), CYP27A1 (P < 0.001), CYP46A1 (P < 0.001) and CH25H (P = 0.046) were all prognostic indicators in Kaplan-Meier survival analysis **(A**–**F)**. And CYP46A1 (HR = 0.893, 95%CI (0.764-0.921), P < 0.001) and CH25H (HR = 0.885, 95%CI (0.825-0.949), P < 0.001) were shown to be independent prognostic factors in multivariable model **(G**–**H)** (Green for univariate and red for multivariate).

Secondly, the overall survival of prognosis of CELF5, GSG1L, AMACR, AKR1D1, CYP27A1, CYP46A1, and CH25H was summarized in **Table S3**. AMACR (P = 0.031), AKR1D1 (P = 0.001), CYP27A1 (P = 0.008), and CYP46A1 (P = 0.024) were significant genes related to prognosis in PROGgeneV2 (**Figure S2**). CELF5 (P = 0.007) and GSG1L (P = 0.006) were significantly related to prognosis in two different datasets of SurvExpres (GSE13041 OS P = 0.002; GSE16011 OS P < 0.001; TCGA GBM OS P = 0.281; TCGA GBM 2016 OS P = 0.002) (**Figure S5**). CYP27A1 (P = 0.022) was significantly related to prognosis in Linkedomics (**Figure S7**). Besides, CELF5 (P = 0.011), AKR1D1 (P = 0.005), and CYP46A1 (P = 0.014) were significantly related to prognosis; integrated genes (P = 0.025) were also significant related to prognosis in the cBioportal database (**Figure S8**). Additionally, analysis based on 1,018 Chinese glioma patients suggested that CELF5 (P < 0.001), GSG1L (P = 0.002), AMACR (P < 0.001), CYP27A1 (P < 0.001), CYP46A1 (P < 0.001), and CH25H (P = 0.046) were all prognostic indicators in Kaplan-Meier survival analysis (**Figures 9A–F**). CYP46A1 (HR = 0.893, 95%CI (0.764-0.921), P < 0.001) and CH25H (HR = 0.885, 95%CI (0.825-0.949), P < 0.001) were shown to be independent prognostic factors in multivariable model (**Figures 9G, H**). The ROC curve of the multivariable model is illustrated in **Figure S12**.

## Discussion

GBM was fatal and invasive. Early diagnosis for the malignant tumor was essential for the OS (Zhou et al., 2016). The development and recurrence might aggravate the tumor and lead to poor prognosis (Alifieris and Trafalis, 2015). Research indicated that biomarkers could be used for prognosis (Zhou et al., 2018a), based on that, we discussed the role of AS in tumorigenesis and prognosis of GBM.

In the current study, the prognostic regulation network of SFs and ASEs was constructed, showing CELF5 positively and negatively regulated GSG1L|35696|AP and GSG1L|35698|AP. Additionally, GSG1L|35696|AP and GSG1L|35698|AP showed significant relationships with cancer status. Eventually, prognostic KEGG pathways were selected by GSVA, and the primary bile acid synthesis pathway was the most significant pathway co-expressed with PSI values of GSG1L|35698|AP, GSG1L|35696|AP. Therefore, we assumed that primary bile acid synthesis might be the downstream pathway of regulatory CELF5 and GSG1L in the prognosis of GBM.

CELF5 interacted with Human cytomegalovirus (HCMV) protein and regulated its genome DNA synthesis (Zou et al., 2018). HCMV participated in tumor regulation like suppressed apoptosis, migration, and invasion (Mcfaline-Figueroa and Wen, 2017; Zou et al., 2018) in GBM. HCMV could regulate the uptake process of glutamate by decreasing the glutamate translocator (Maurice, 1970).

GSG1L was inactive in the AMPA-type glutamate receptors (AMPARs) in cancer (Gu et al., 2016; Andrew et al., 2017). In addition, GBM could secrete glutamate and trigger the AMPARs, leading to cell death in neurons surrounding the tumor (Van Vuurden et al., 2009). What's more, cancer cells in glioma could form synapses with other neurons and communicate by the AMPAR, improving proliferation and growth of glioma (Venkataramani et al., 2019; Venkatesh et al., 2019).

Up to now, there has been no publication focused on the direct regulation of CELF5 and GSG1L. We detected that they are all involved in the glutamate-related processes like cell apoptosis and tumor proliferation based on reports up to date.

Some studies indicated that bile acid and its derivatives were related to several cancers, like multiple myeloma, hepatoma, GBM, and colon cancer, and bile acid could control the development of cancer by its cytotoxicity to cancer and by signaling to immune cells (Brossard et al., 2010; Ma et al., 2018).

Further, in bile acid synthesis, some products of bile acid regulated the PI3K dependent Bad pathway, causing apoptosis of neurons by glutamate(Castro et al., 2004). As we mentioned before, CELF5 could interacted with HCMV, which could regulate the translocator, and GSG1L could suppress the AMPAR. Although there was no such report of the relationship of CELF5, GSG1L, and primary acid bile synthesis, glutamate-related apoptosis might be the link.

Overall, CELF5 was the remarkable SF and GSG1L was the remarkable OS-SE related to status. CELF5 significantly regulated GSG1L. Additionally, primary bile acid was the candidate pathway in the downstream of the regulation between CELF5 and GSG1L. However, because our analysis was only an *in silico* analysis, it was limited by the sample size and support of the molecular mechanism. However, to minizine the systemic bias, we applied multidimension validation for CELF5, GSG1L, and primary bile acid synthesis based on multiple online databases, showing the stability of the results of this study.

With the aim of making our hypothesis more scientific, a convincing and high-level basic experimental verification will be launched in the future. Based on some studies devoted to ASEs, such as ASEs in pan-cancer, pancreatic cancer, and Renal Cancer (Calabretta et al., 2016; Qi et al., 2016; Chen et al., 2017; Couture et al., 2017). All genes in our hypothesis will be detected in a large quantity of clinical samples (tumor vs healthy; tumor vs adjacent normal tissue) by IHC to explore the differential expression. A direct mechanism between CELF5 and GSG1L will be validated by Co-immunoprecipitation and RNA immunoprecipitation. Furthermore, an engineered splicing factor will be designed to validate the splicing pattern which produces a certain splicing isoform of GSG1L. Besides, immunofluorescence staining will be utilized to explore the location of CELF5 and GSG1L. What’s more, the positive or negative regulatory relationship among CELF5, splicing isoforms of GSG1L, primary bile acid synthesis signaling pathway, and GBM tumorigenesis will be validated by biological function assays like gain/loss of function and rescue assays. These function and direct mechanism assays might offer more evidence for these potential therapeutic targets and novel prognostic factors in GBM.

## Conclusion

Based on the comprehensive bioinformatics analysis, we proposed that aberrant splicing factor CUGBP Elav-like family member 5 (CELF5) positively and negatively regulated ASE of GSG1L and the primary bile acid synthesis pathway might play an important role in tumorigenesis and prognosis of GBM. The scientific hypothesis might offer direction for subsequent biological experiments.

## Data Availability Statement

All datasets used for this study are available from the TCGA-GBM program.

## Ethics Statement

This study was approved by the Ethics Committee of Tongji University School of Medicine.

## Author Contributions

Conception/design: RH, ZL, CL, GW, PY, LP, JW, XZ, PH, JZ, ZC, ZH, LC, and JiZ. Provision of study material: ZH, LC, and JiZ. Collection and/or assembly of data: RH, ZL, CL, GW, PY, LP, JW, XZ, PH, JZ and ZC. Data analysis and interpretation: RH, ZL, CL, GW, PY, LP, JW, XZ, PH, JZ and ZC. Manuscript writing: RH, ZL, CL, GW, PY, LP, JW, XZ, PH, JZ, ZC, ZH, LC, and JiZ. Final approval of manuscript: RH, ZL, CL, GW, PY, LP, JW, XZ, PH, JZ, ZC, ZH, LC, and JiZ.

## Funding

This study was supported in part by the National Natural Science Foundation of China (No. 81501203), Shanghai Municipal Health Commission (No.201940306) and Henan medical science and technology research project (No. 201602031).

## Conflict of Interest

The authors declare that the research was conducted in the absence of any commercial or financial relationships that could be construed as a potential conflict of interest.

## Abbreviation

GSG1L, Germ Cell-Specific Gene 1-Like Protein; CELF5, CUGBP Elav-like family member 5; GBM, Glioblastoma Multiforme; WHO, World Health Organization; TCGA, The Cancer Genome Atlas; OS-SEs, overall-survival-associated splicing events; SF, splicing factor; OS, overall survival; ASE, Alterative splicing events; AS, Alternative splicing; ME, Mutually exons; AD, Alternate donor site; RI, Retained intron; AA, Alternate acceptor site; AP, Alternate promoter; AT, Alternate terminator; ES, Exon skip; PSI, percent spliced in; KEGG, Kyoto Encyclopedia of Genes and Genomes; GSVA, Gene Set Variation Analysis; CCLE, Cancer Cell Line Encyclopedia; GEPIA, Gene Expression Profilling Interactive Analysis; GTEx, Genotype-Tissue Expression; ROC, The receiver operator characteristic; AUC, area under the curve; AMPARs, AMPA-type glutamate receptors; HCMV, Human cytomegalovirus.
