# Supplementary figures and images for: Germ Cell-Specific Gene 1-Like Protein Regulated by Splicing Factor CUGBP Elav-Like Family Member 5 and Primary Bile Acid Biosynthesis are Prognostic in Glioblastoma Multiforme
Source: Front Genet. 2020 Feb 4;10:1380. doi: 10.3389/fgene.2019.01380 (PMC7010853; doi:10.3389/fgene.2019.01380)

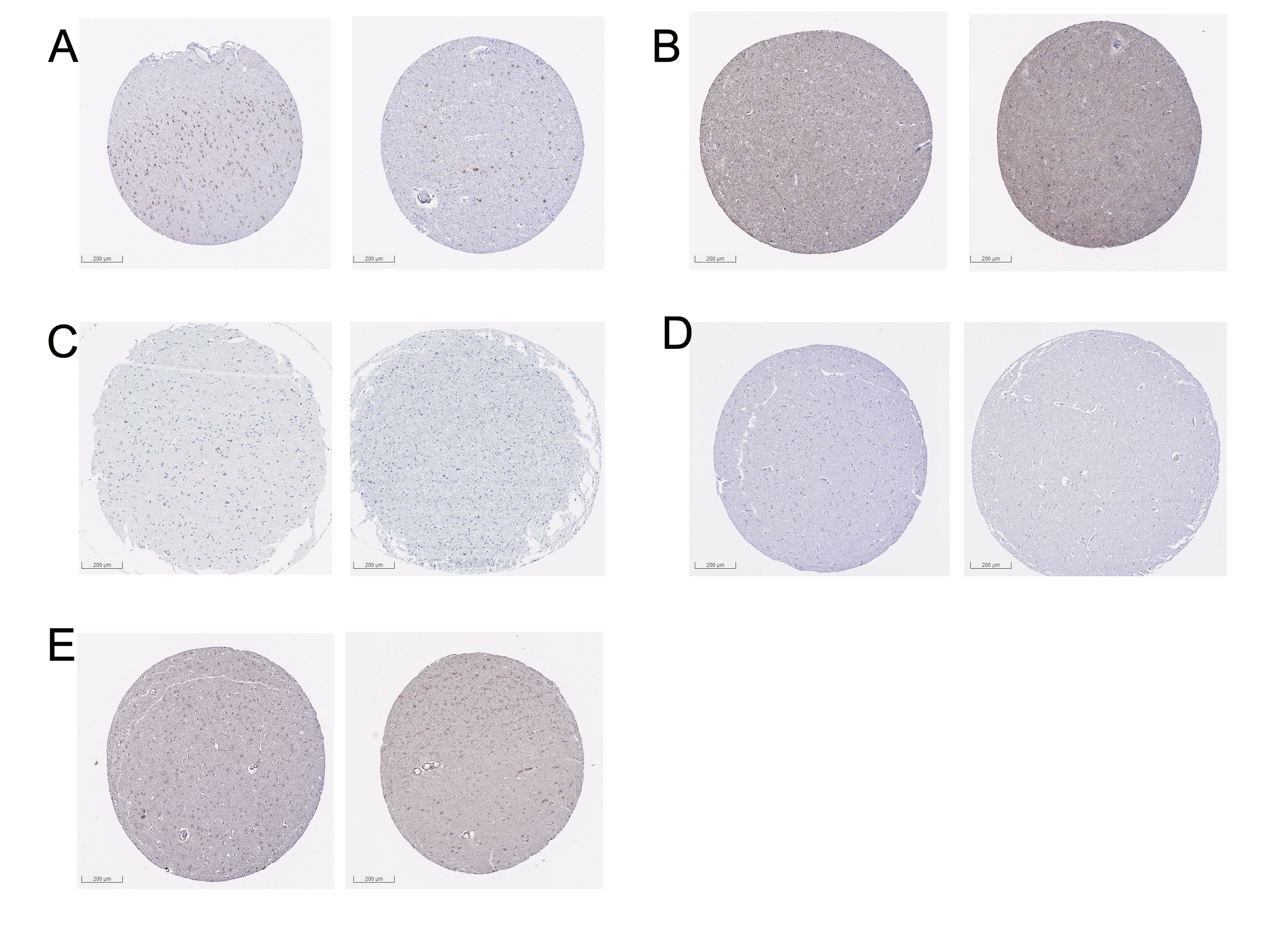

Supplement: Supplementary file 1 [file Image_1.tif]

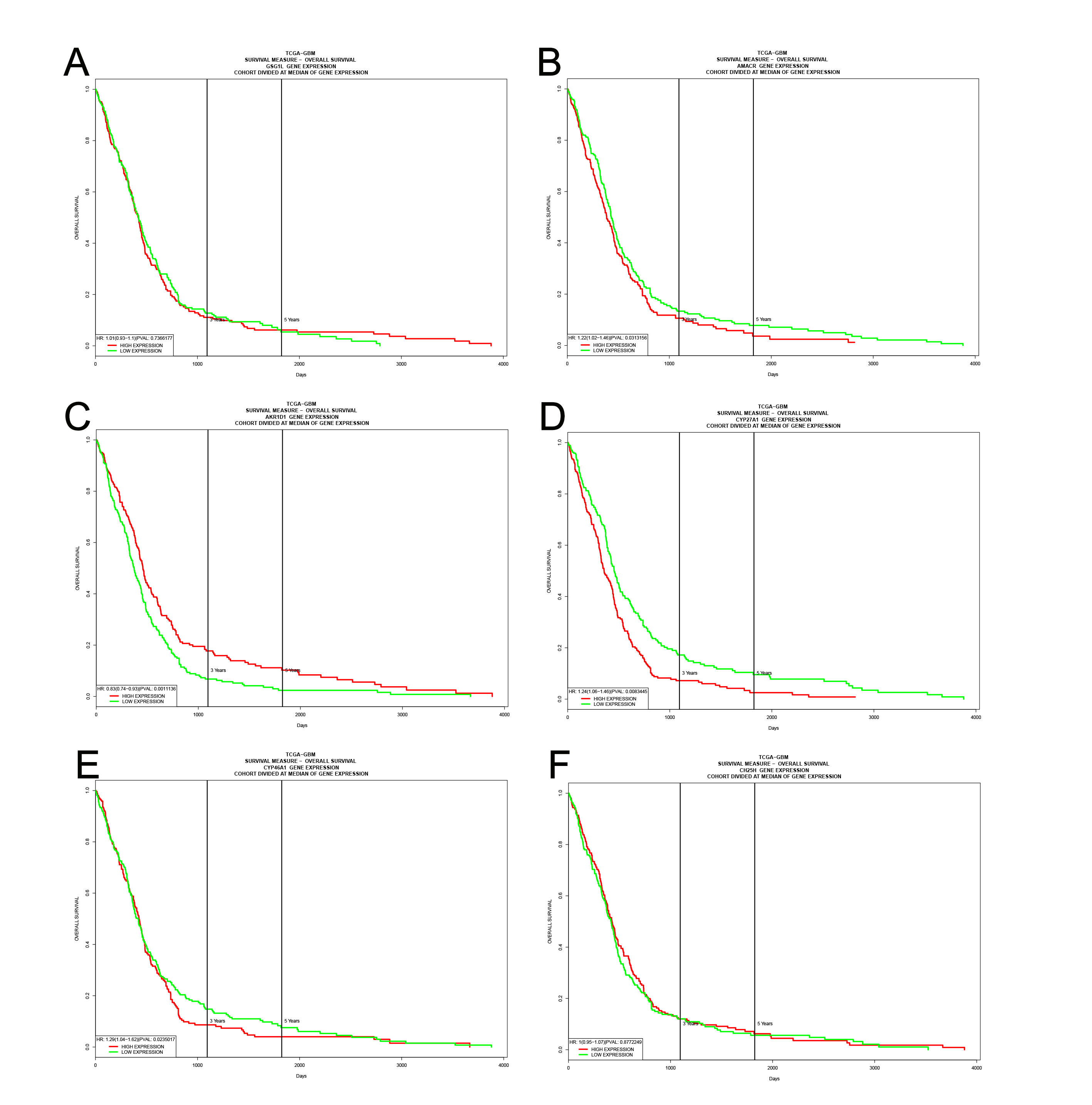

Supplement: Supplementary file 2 [file Image_2.tif]

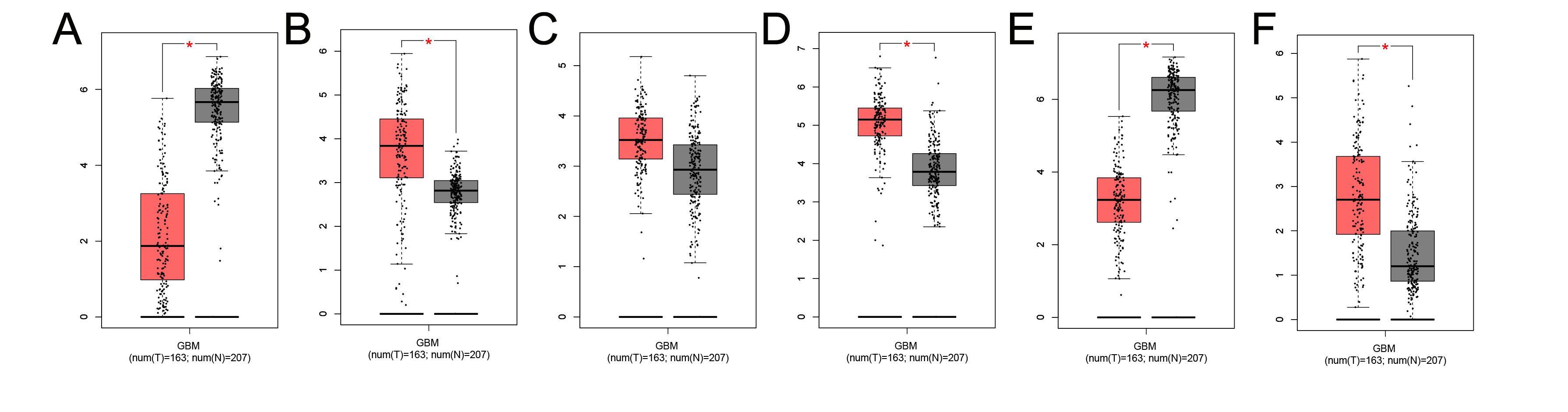

Supplement: Supplementary file 3 [file Image_3.tif]

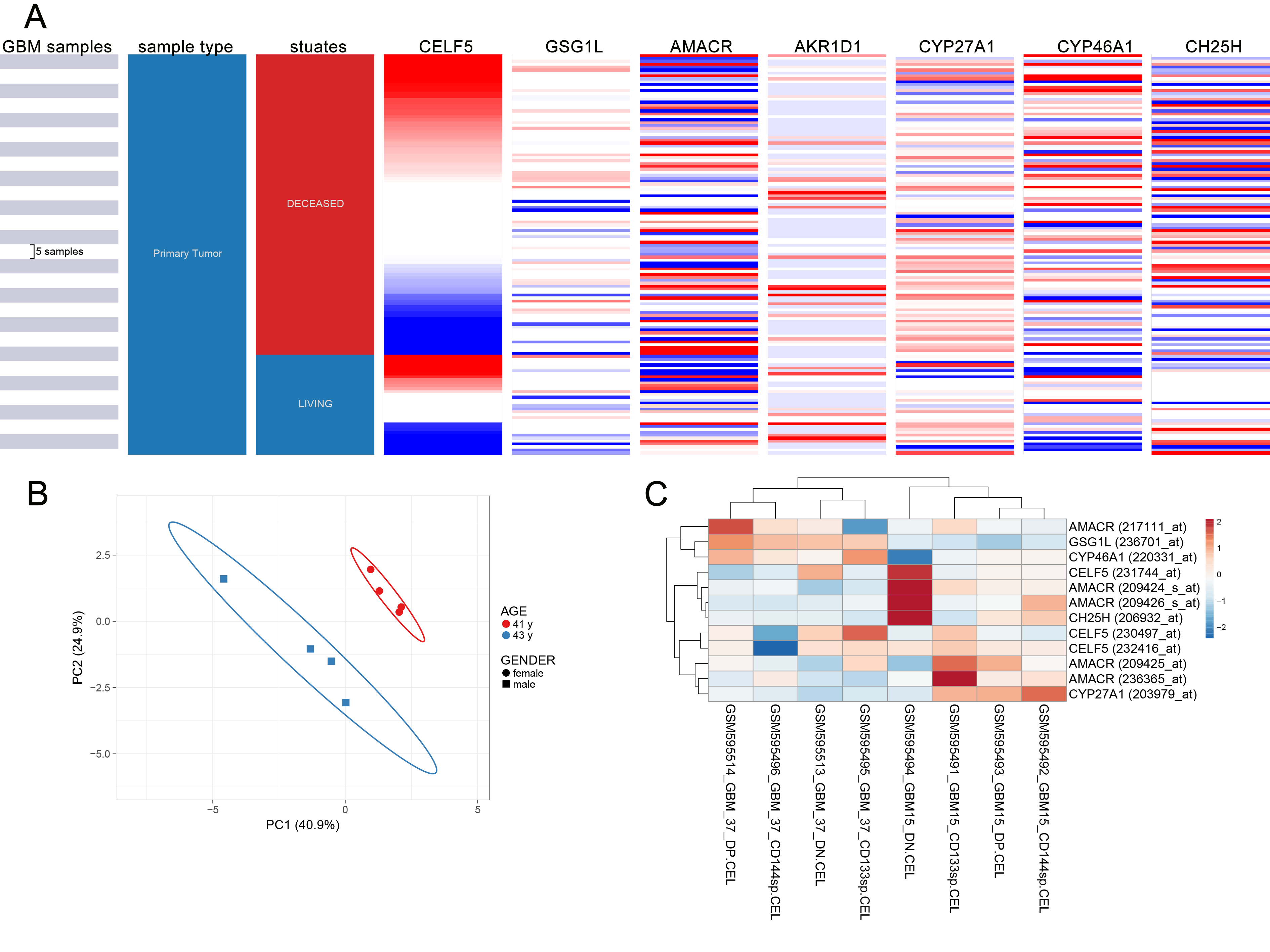

Supplement: Supplementary file 4 [file Image_4.tif]

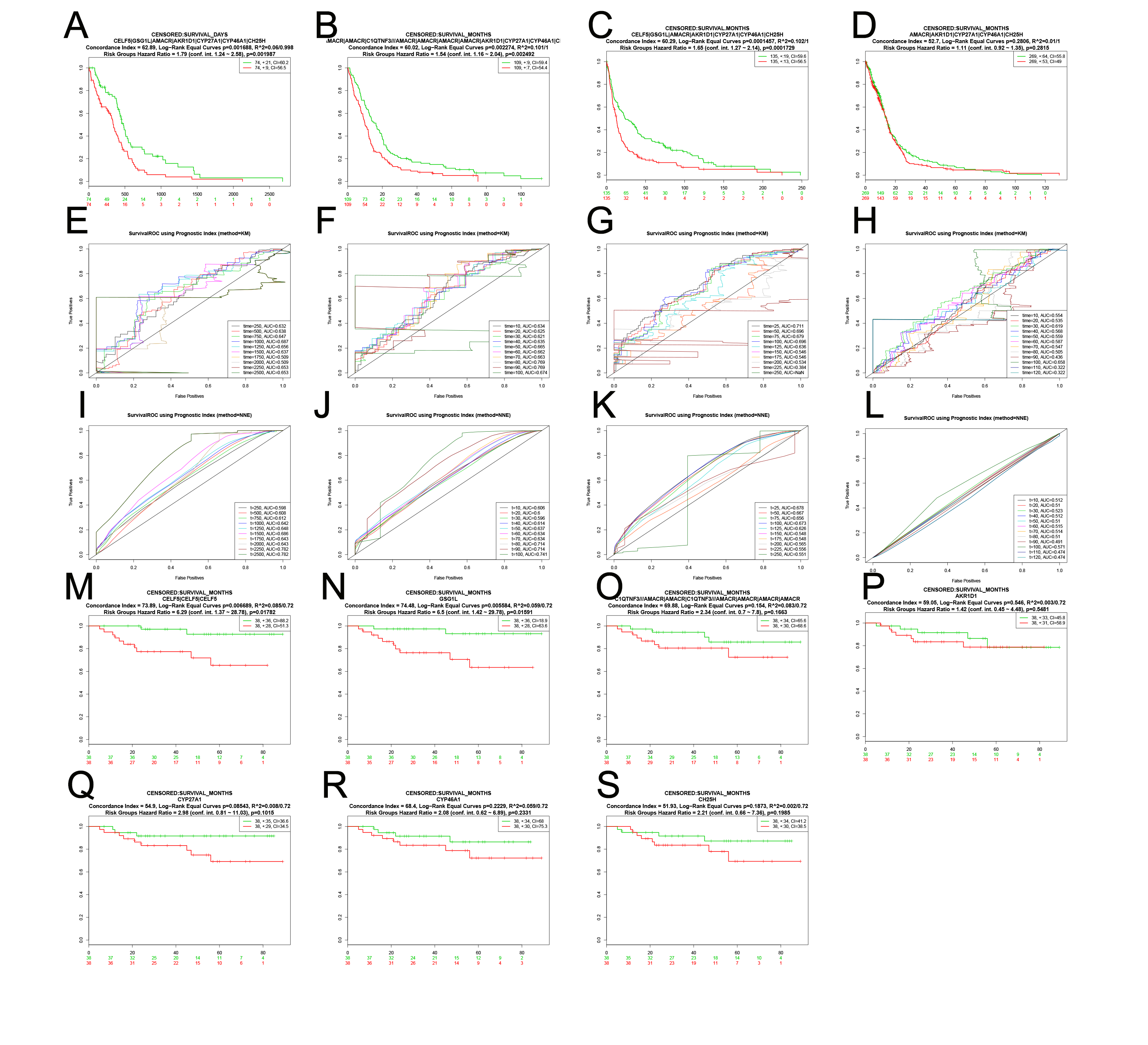

Supplement: Supplementary file 5 [file Image_5.tif]

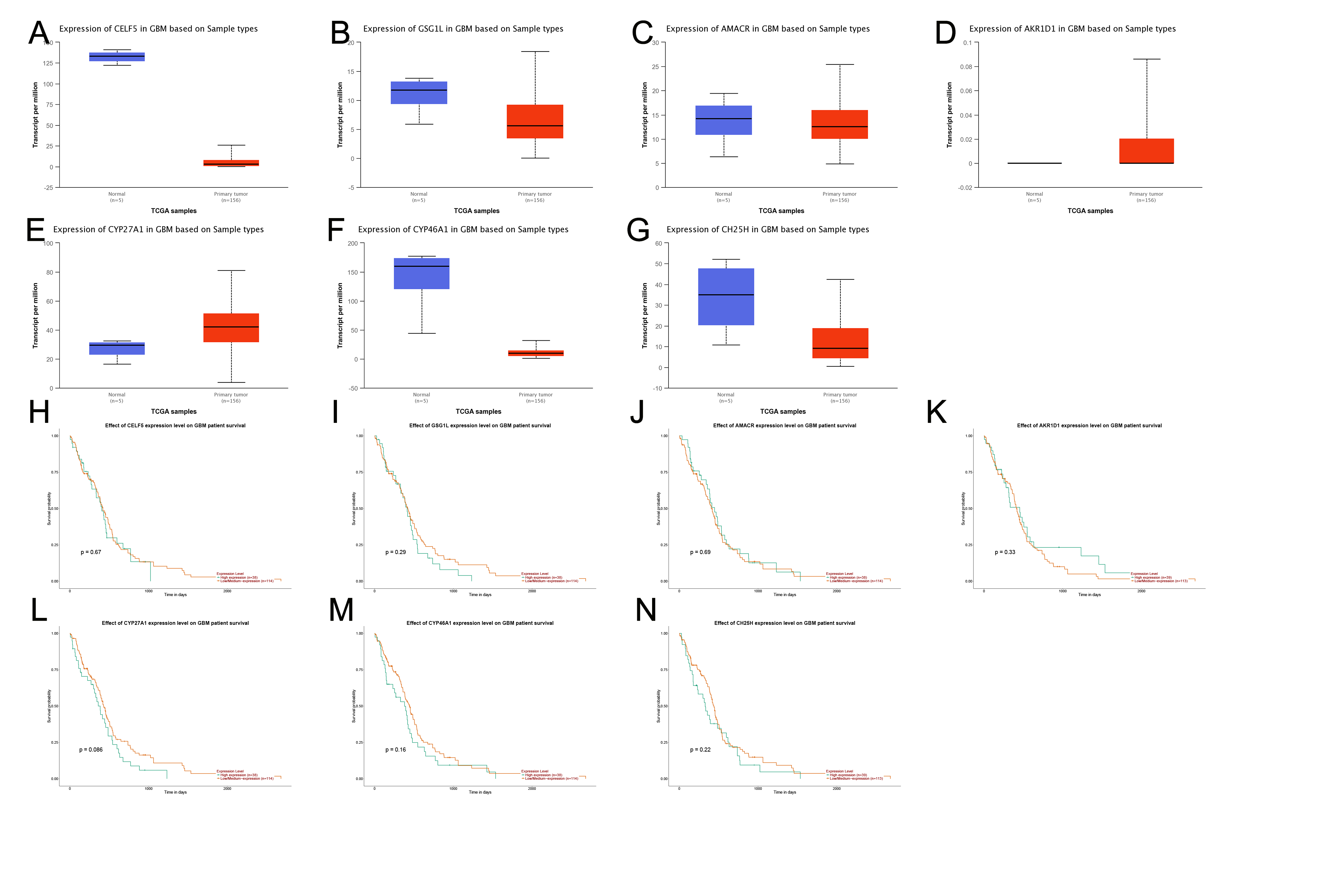

Supplement: Supplementary file 6 [file Image_6.tif]

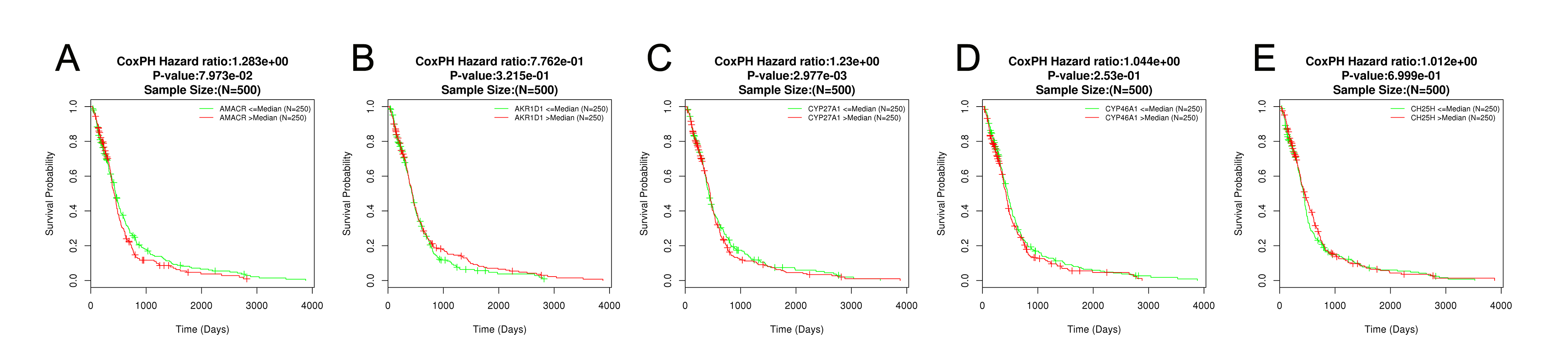

Supplement: Supplementary file 7 [file Image_7.tif]

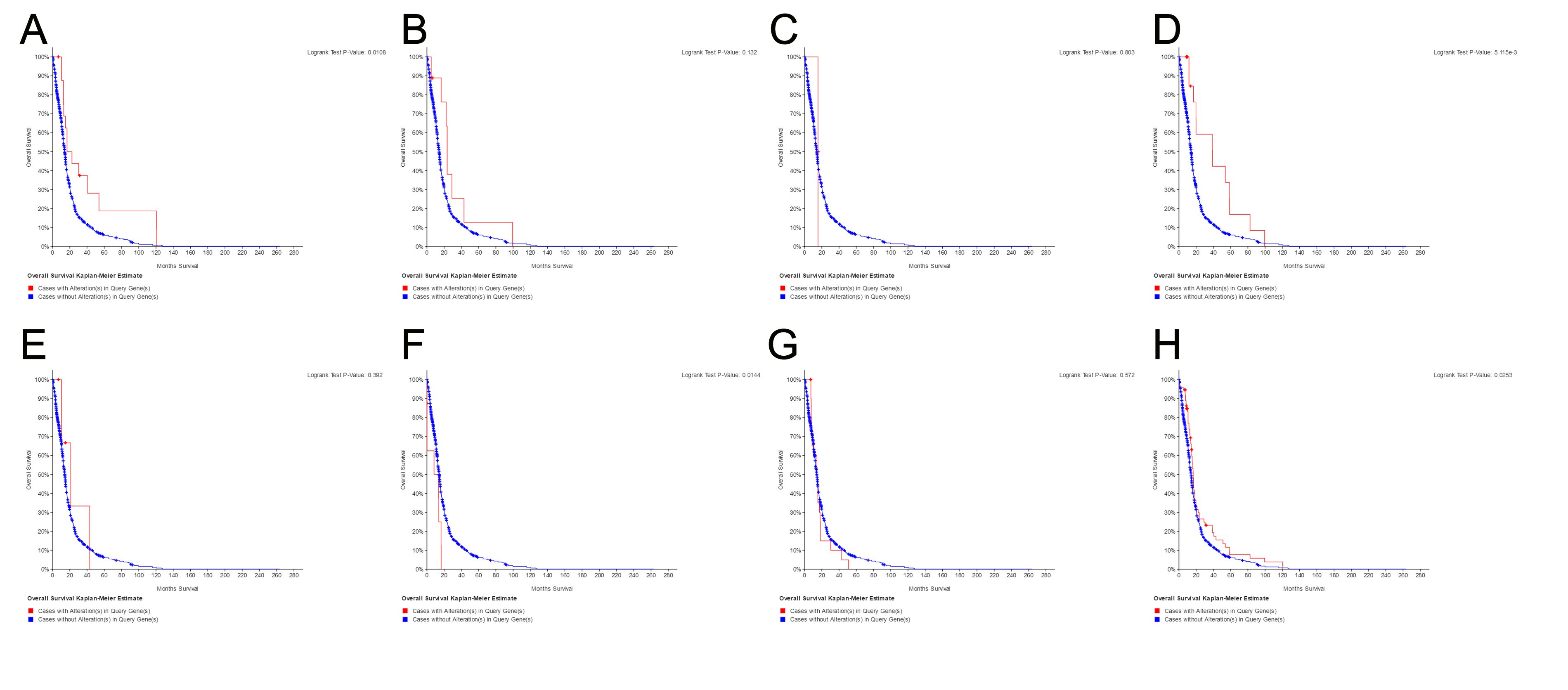

Supplement: Supplementary file 8 [file Image_8.tif]

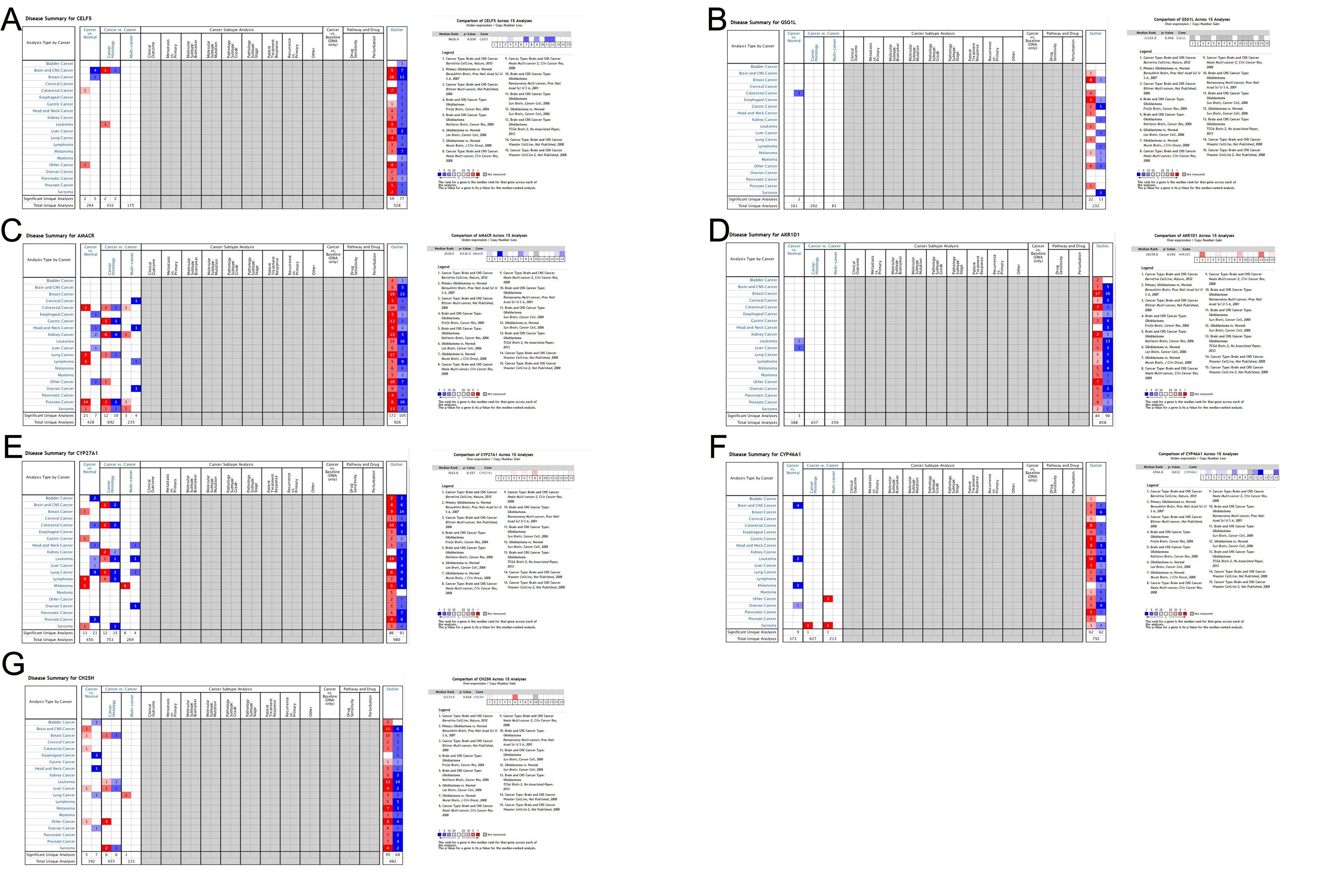

Supplement: Supplementary file 9 [file Image_9.tif]

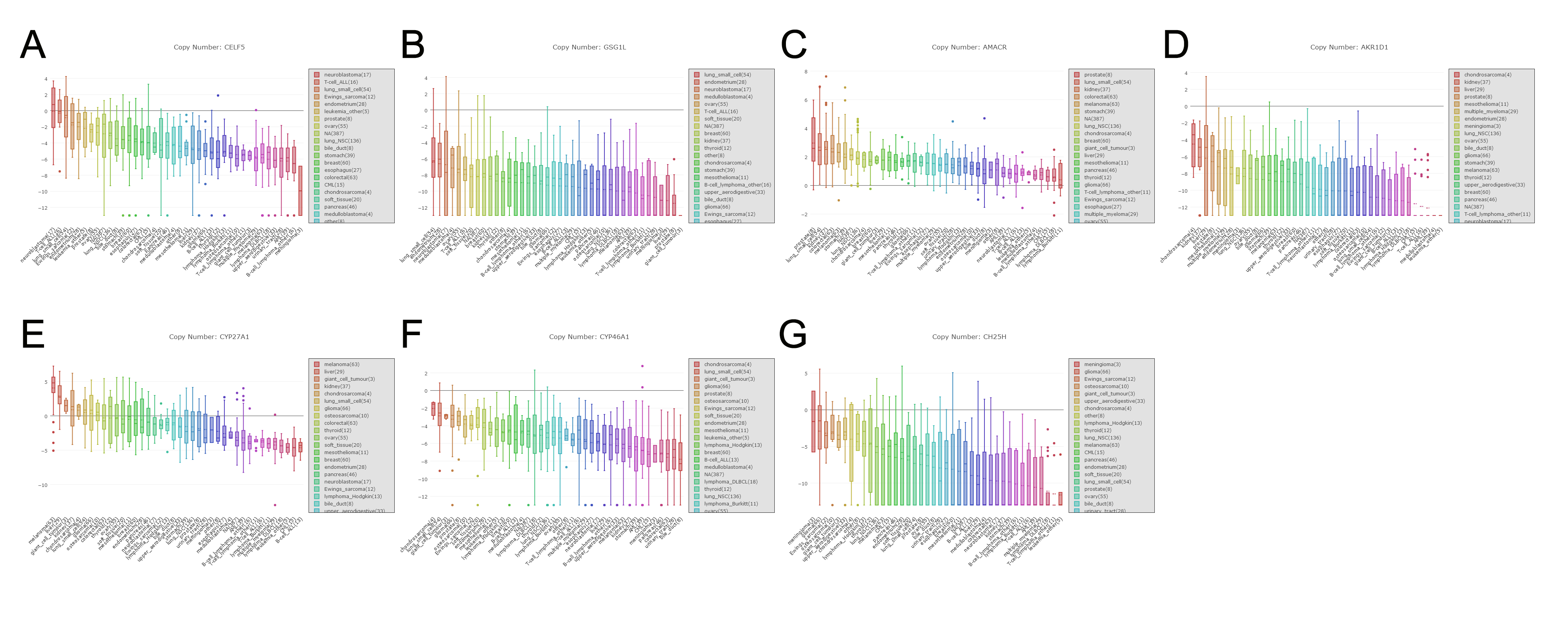

Supplement: Supplementary file 10 [file Image_10.tif]

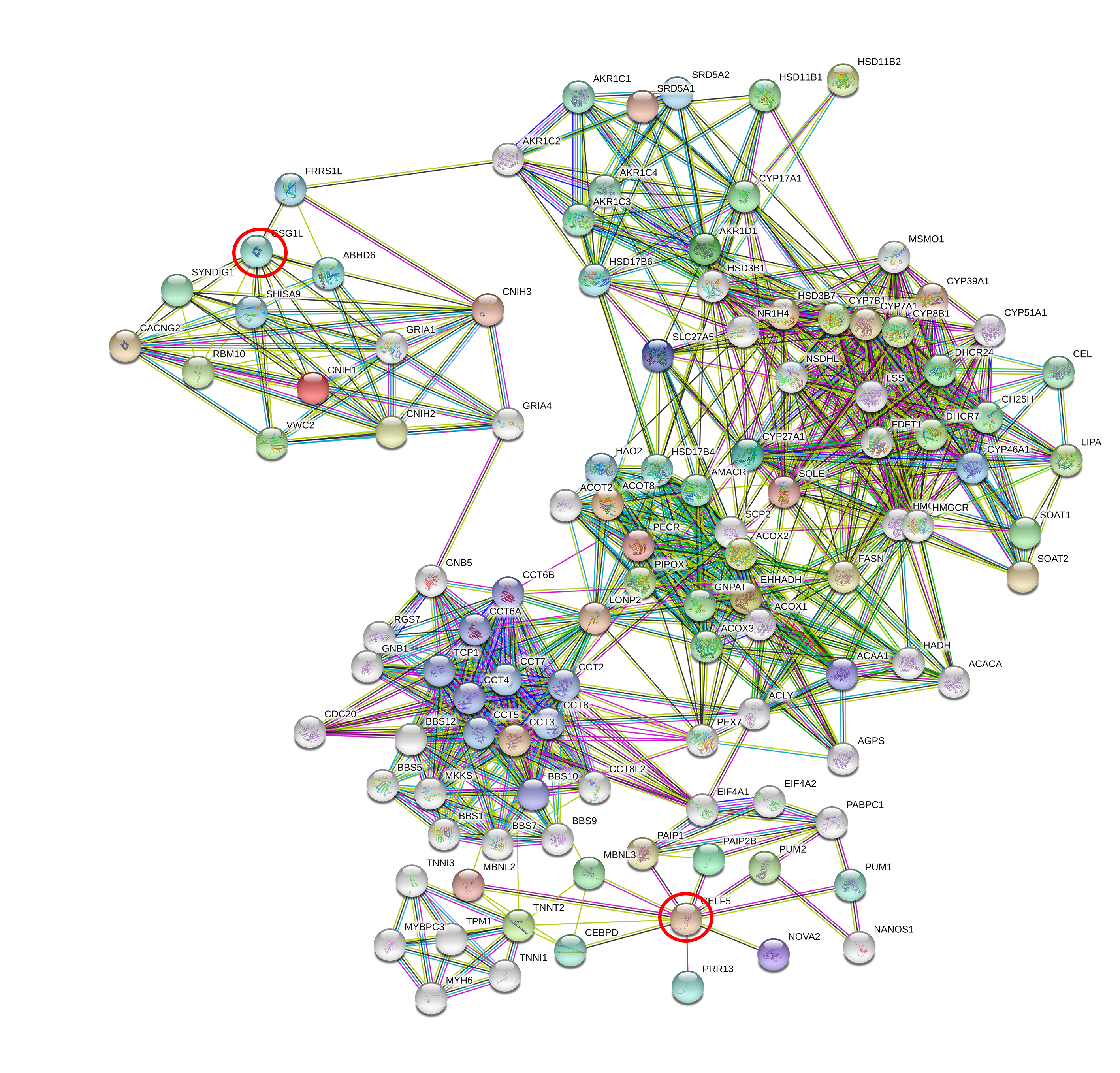

Supplement: Supplementary file 11 [file Image_11.tif]
