# Supplementary material for: Germ Cell-Specific Gene 1-Like Protein Regulated by Splicing Factor CUGBP Elav-Like Family Member 5 and Primary Bile Acid Biosynthesis are Prognostic in Glioblastoma Multiforme
Source: Front Genet. 2020 Feb 4;10:1380. doi: 10.3389/fgene.2019.01380 (PMC7010853; doi:10.3389/fgene.2019.01380)
Supplement: Supplementary file 12 [file Table_1.docx]

**Table S1.** Mutual Exclusivity of CELF5, GSG1L, AMACR, AKR1D1, CYP27A1, CYP46A1 and CH25H in cBioportal database.

| **A** | **B** | **Neither** | **A Not B** | **B Not A** | **Both** | **Log2 Odds Ratio** | **p-Value** | **q-Value** | **Tendency** |
| --- | --- | --- | --- | --- | --- | --- | --- | --- | --- |
| GSG1L | CYP27A1 | 1094 | 6 | 3 | 2 | >3 | <0.001 | 0.01 | Co-occurrence |
| CELF5 | GSG1L | 1088 | 9 | 6 | 2 | >3 | 0.002 | 0.026 | Co-occurrence |
| CELF5 | CYP27A1 | 1090 | 10 | 4 | 1 | >3 | 0.049 | 0.292 | Co-occurrence |
| CYP27A1 | CH25H | 1087 | 4 | 13 | 1 | >3 | 0.062 | 0.292 | Co-occurrence |
| GSG1L | AKR1D1 | 1088 | 7 | 9 | 1 | >3 | 0.07 | 0.292 | Co-occurrence |
| CELF5 | AKR1D1 | 1085 | 10 | 9 | 1 | >3 | 0.096 | 0.292 | Co-occurrence |
| GSG1L | CH25H | 1084 | 7 | 13 | 1 | >3 | 0.097 | 0.292 | Co-occurrence |
| CELF5 | CH25H | 1081 | 10 | 13 | 1 | >3 | 0.131 | 0.345 | Co-occurrence |
| AKR1D1 | CH25H | 1081 | 10 | 14 | 0 | <-3 | 0.88 | 1 | Mutual exclusivity |
| CYP46A1 | CH25H | 1086 | 5 | 14 | 0 | <-3 | 0.938 | 1 | Mutual exclusivity |
| CELF5 | CYP46A1 | 1089 | 11 | 5 | 0 | <-3 | 0.951 | 1 | Mutual exclusivity |
| AKR1D1 | CYP27A1 | 1090 | 10 | 5 | 0 | <-3 | 0.955 | 1 | Mutual exclusivity |
| AKR1D1 | CYP46A1 | 1090 | 10 | 5 | 0 | <-3 | 0.955 | 1 | Mutual exclusivity |
| GSG1L | CYP46A1 | 1092 | 8 | 5 | 0 | <-3 | 0.964 | 1 | Mutual exclusivity |
| CYP27A1 | CYP46A1 | 1095 | 5 | 5 | 0 | <-3 | 0.978 | 1 | Mutual exclusivity |
| AMACR | AKR1D1 | 1095 | 0 | 10 | 0 | >3 | 1 | 1 | Co-occurrence |
| CELF5 | AMACR | 1094 | 11 | 0 | 0 | >3 | 1 | 1 | Co-occurrence |
| GSG1L | AMACR | 1097 | 8 | 0 | 0 | >3 | 1 | 1 | Co-occurrence |
| AMACR | CYP27A1 | 1100 | 0 | 5 | 0 | >3 | 1 | 1 | Co-occurrence |
| AMACR | CYP46A1 | 1100 | 0 | 5 | 0 | >3 | 1 | 1 | Co-occurrence |
| AMACR | CH25H | 1091 | 0 | 14 | 0 | >3 | 1 | 1 | Co-occurrence |
