# Supplementary material for: Germ Cell-Specific Gene 1-Like Protein Regulated by Splicing Factor CUGBP Elav-Like Family Member 5 and Primary Bile Acid Biosynthesis are Prognostic in Glioblastoma Multiforme
Source: Front Genet. 2020 Feb 4;10:1380. doi: 10.3389/fgene.2019.01380 (PMC7010853; doi:10.3389/fgene.2019.01380)
Supplement: Supplementary file 13 [file Table_2.docx]

**Table S2** Summary of multidimensional external validation results of gene expression base on multiple databases

|  | **CELF5** | | **GSG1L** | | **AMACR** | | **AKR1D1** | | **CYP27A1** | | **CYP46A1** | | **CH25H** | | **Results** |
| --- | --- | --- | --- | --- | --- | --- | --- | --- | --- | --- | --- | --- | --- | --- | --- |
|  | **N** | **G** | **N** | **G** | **N** | **G** | **N** | **G** | **N** | **G** | **N** | **G** | **N** | **G** |  |
| **The human protein atlas** | **↑** | **NA** | **↑** | **NA** | **ND** | **NA** | **ND** | **NA** | **↑** | **NA** | **NA** | **NA** | **NA** | **NA** | CELF5, GSG1L and CYP27A1 high-expressed in normal cerebral cortex in brain (Figure S1). |
| **GTEx** | **↑** | **NA** | **↓** | **NA** | **↓** | **NA** | **ND** | **NA** | **-** | **NA** | **↑** | **NA** | **↓** | **NA** | CELF5 and CYP46A1 high-expressed, while AMACR and CH25H low-expressed in normal cerebral cortex in brain (Figure S2). |
| **PROGgeneV2** | **NA** | **NA** | **NA** | **-** | **NA** | **↑** | **NA** | **↓** | **NA** | **↑** | **NA** | **↑** | **NA** | **-** | AMACR, CYP27A1 and CYP46A1 high-expressed, while AKR1D1 low-expressed in tumor tissue (Figure S3). |
| **GEPIA** | **↑** | **↓** | **↓** | **↑** | **↓** | **↑** | **↓** | **↓** | **↓** | **↑** | **↑** | **↓** | **↓** | **↑** | CELF5 and CYP46A1 high-expressed in normal tissue and low-expressed in tumor tissue; GSG1L, AMACR, CYP27A1 and CH25H low-expressed in normal tissue and high-expressed in tumor; AKR1D1 low-expressed in normal and tumor tissue (Figure S4). |
| **Express altas** | **NA** | **↓** | **NA** | **-** | **NA** | **NA** | **NA** | **↑** | **NA** | **-** | **NA** | **↓** | **NA** | **↓** | CELF5, CYP46A1 and CH25H low-expressed in tumor tissue; AKR1D1 high-expressed in tumor tissue. |
| **UCSC xena** | **NA** | **-** | **NA** | **↑** | **NA** | **↑** | **NA** | **↓** | **NA** | **↑** | **NA** | **-** | **NA** | **-** | GSG1L, AMACR and CYP27A1 high-expressed in tumor tissue; AKR1D1 low-expressed in tumor tissue (Figure S5). |
| **SurvExpress** | **NA** | **↑** | **NA** | **-** | **NA** | **-** | **NA** | **-** | **NA** | **↑** | **NA** | **↑** | **NA** | **↑** | CELF5, CYP27A1, CYP46A1 and CH25H high-expressed in tumor tissue (Figure S6). |
| **UALCAN** | **↑** | **↓** | **↑** | **-** | **-** | **-** | **↓** | **↑** | **↓** | **↑** | **↑** | **↓** | **↑** | **↓** | CELF5, CYP46A1 and CH25H high-expressed in normal tissue and low-expressed in tumor tissue; AKR1D1 and CYP27A1 low-expressed in normal tissue and high-expressed in tumor; GSG1L high-expressed in normal tissue (Figure S7). |
| **Linkedomics** | **NA** | **NA** | **NA** | **NA** | **NA** | **↑** | **NA** | **↓** | **NA** | **↑** | **NA** | **↑** | **NA** | **-** | AMACR, CYP27A1 and CYP46A1 high-expressed in tumor tissue; AKR1D1 low-expressed in tumor tissue (Figure S8). |
| **cBioportal** | **NA** | **-** | **NA** | **↑** | **NA** | **↑** | **NA** | **↓** | **NA** | **↑** | **NA** | **↑** | **NA** | **↑** | GSG1L, AMACR, CYP27A1, CYP46A1 and CH25H high-expressed in tumor tissue; AKR1D1 low-expressed in tumor tissue (Figure S9). |
| **Oncomine** | **NA** | **↓** | **NA** | **-** | **NA** | **↓** | **NA** | **↑** | **NA** | **↑** | **NA** | **↓** | **NA** | **↑** | CELF5, AMACR and CYP46A1 low expressed in tumor tissue; AKR1D1 and CYP27A1 high-expressed in tumor tissue (Figure S10). |
| **CCLE** | **NA** | **↓** | **NA** | **-** | **NA** | **↑** | **NA** | **-** | **NA** | **↑** | **NA** | **↑** | **NA** | **↑** | CELF5 low-expressed in tumor cell line; AMACR, CYP27A1, CYP46A1 and CH25H high-expressed in tumor cell line (Figure S11). |

Note: “N” was defined as normal; “G” was defined as Glioblastoma multiforme;“↑” was defined as a significantly high-expressed gene; “↓” was defined as a significantly low-expressed gene; “NA” was defined as “Not available”; “ND” was defined as “Not detached”; “-” was defined as a gene with no significant difference in expression.

Abbreviations: GBM, Glioblastoma Multiforme; GTEx, Genotype-Tissue Expression; CCLE, Cancer Cell Line Encyclopedia; GEPIA, Gene Expression Profilling Interactive Analysis.
