# Supplementary material for: Germ Cell-Specific Gene 1-Like Protein Regulated by Splicing Factor CUGBP Elav-Like Family Member 5 and Primary Bile Acid Biosynthesis are Prognostic in Glioblastoma Multiforme
Source: Front Genet. 2020 Feb 4;10:1380. doi: 10.3389/fgene.2019.01380 (PMC7010853; doi:10.3389/fgene.2019.01380)
Supplement: Supplementary file 14 [file Table_3.docx]

**Table S3** Summary of multidimensional external validation results of prognosis based on multiple databases

|  | **CELF5** | **GSG1L** | **AMACR** | **AKR1D1** | **CYP27A1** | **CYP46A1** | **CH25H** | **Results** |
| --- | --- | --- | --- | --- | --- | --- | --- | --- |
| **PROGgeneV2** | NA | OS  P = 0.737 | OS  P = 0.031 | OS  P = 0.001 | OS  P = 0.008 | OS  P = 0.024 | OS  P = 0.877 | AMACR, AKR1D1, CYP27A1 and CYP46A1 are significant related to prognosis (Figure S3). |
| **SurvExpress** | GSE37418OS P = 0.007 | GSE37418 OS P = 0.006 | GSE37418 OS P = 0.154 | GSE37418 OS P = 0.546 | GSE37418 OS P = 0.085 | GSE37418 OS P = 0.223 | GSE37418 OS P = 0.187 | CELF5 and GSG1L are significant related to prognosis; integrated genes are significant related to prognosis (Figure S6). |
|  | GSE13041 OS P = 0.002  GSE16011 OS P < 0.001  TCGA GBM OS P = 0.281  TCGA GBM 2016 OS P = 0.002 | | | | | | |  |
| **Linkedomics** | NA | NA | OS P = 0.080 | OS P = 0.322 | OS P = 0.022 | OS P = 0.253 | OS P = 0.700 | CYP27A1 is significant related to prognosis (Figure S8). |
| **cBioportal** | OS P = 0.011 | OS P = 0.132 | OS P = 0.803 | OS P = 0.005 | OS P = 0.392 | OS P = 0.014 | OS P = 0.672 | CELF5, AKR1D1 and CYP46A1 are significant related to prognosis; integrated genes are also significant related to prognosis (Figure S9). |
|  | OS of integrated genes P = 0.025 | | | | | | |  |

Note: OS, Overall survival; NA, not available.
